# CXXC finger protein 1 (CFP1) bridges the reshaping of genomic H3K4me3 signature to the advancement of lung adenocarcinoma

**DOI:** 10.1038/s41392-023-01612-3

**Published:** 2023-09-21

**Authors:** Tao Fan, Chu Xiao, Hengchang Liu, Yu Liu, Liyu Wang, He Tian, Chunxiang Li, Jie He

**Affiliations:** 1 Department of Thoracic Surgery, National Cancer Center/National Clinical Research Center for Cancer/Cancer Hospital, Chinese Academy of Medical Sciences and Peking Union Medical College, Beijing, 100021 China; 2 Department of Colorectal Surgery, National Cancer Center/National Clinical Research Center for Cancer/Cancer Hospital, Chinese Academy of Medical Sciences and Peking Union Medical College, Beijing, China; 3 Department of Intervention, National Cancer Center/National Clinical Research Center for Cancer/Cancer Hospital, Chinese Academy of Medical Sciences and Peking Union Medical College, Beijing, China

**Keywords:** Lung cancer, Genome informatics

## Abstract

Histone H3 lysine 4 trimethylation (H3K4me3) is a canonical chromatin modification associated with active gene transcription, playing a pivotal role in regulating various cellular functions. Components of the H3K4me3 methyltransferase complex, known as the proteins associated with SET1 (COMPASS), have been implicated in exerting cancer-protective or cancer-inhibitory effects through inducive H3K4me3 modification. However, the role of the indispensable non-catalytic component of COMPASS CXXC-type zinc finger protein 1 (CFP1) in malignant progression remains unclear. We have unveiled that CFP1 promote lung adenocarcinoma (LUAD) cell proliferation, migration, and invasion while impairing cell apoptosis through in vitro and in vivo models. In addition, high CFP1 expression was identified as emerged as an adverse prognostic indicator across multiple public and in-house LUAD datasets. Notably, CFP1 deficiency led to dual effects on cancer cell transcriptome including extensive inactivation of cancer-promoting as well as activation of cancer repressors. Combining this with the chromatin immunoprecipitation sequencing (ChIP-seq) analysis, we showed that CFP1 ablation reshaped the genomic H3K4me3 distribution signature, with prominent effects on TGF-β and WNT signaling pathways. Collectively, our study proposes that CFP1 mediates tumorigenesis by genomic histone methylation reprogramming, offering insights for future investigations into epigenetic modifications in cancer progression and potential therapeutic advancements.

## Introduction

Lung cancer is the leading cause of cancer-related deaths around the world.^1^ Although considerable development in therapeutic strategies has been achieved, the 5-year survival rate of lung cancer patients is still very low.^2^ Therefore, it is imperative to seek potential oncogenic targets for advancing therapeutic development.

Epigenetic regulation in cancer constantly captures wide attention due to its universality and reversibility in cancer progressions, as well as the numerous potential targets that can be druggable. For example, the application of DNA methyltransferase inhibitors and histone deacetylase inhibitors has been approved in hematological malignancy treatments.^3^ In addition, histone methylation-related target drugs, such as Tazemetostat, an EZH2 inhibitor repressing histone H3 lysine 27 methylation was effective in treating epithelioid sarcoma.^3,4^ H3K4me3 is one of the fundamental types of histone modification, mediating transcription initiation and elongation,^5,6^ and has been reported to play vital roles in biological processes including cell development, differentiation, and malignant diseases.^6–8^ Its methyltransferases in charge are associated with the progression of several cancer types. The catalytic subunit of COMPASS, SETD1B, which mediates H3K4 trimethylation in mammals has been classified as a Tier 2 cancer gene, associated with gastric cancer, colorectal cancer, and clear cell renal cell carcinoma. SETD1A is also reported to play a tumor-promoting role in gastric cancer.^9^ The non-catalytic subunits like WDR5 also had been reported to engage in the cancer process.^10^ CFP1 is indispensable for H3K4 trimethylation, and CSP40 deficiency (CFP1 yeast homolog) in yeast will lead to a global loss of H3K4me3.^11^ However, the knowledge of how CFP1 plays in cancer is absent.

CFP1 is a multifunctional protein with several functional domains.^12^ The SET1 Interaction Domain (SID) is required for CFP1 interaction with the H3K4 methyltransferases SETD1A and SETD1B.^9^ CXXC is a DNA binding domain that specifically binds to unmethylated CpG dinucleotides, avoiding the SETD1A and SETD1B localizing to regions outside of CpG islands (CGIs), thereby preventing ectopic H3K4me3 peaks from appearing on inappropriate chromatin compartments.^13,14^ In addition, CFP1 interacts with DNMT1 to facilitate cytosine methylation.^13^ CFP1 is required in various biological processes, including spermatogenesis, meiotic cell cycle progression in mouse oocytes, follicle growth, and ovulation.^9,15,16^ Loss of CFP1 will lead to murine embryonic peri-implantation lethality and the impairment of normal hematopoiesis in animal experiments.^9^ Given the essential role of CFP1 in biological processes and the extensive regulatory effects of H3K4me3 on tumorigenesis, we ask whether CFP1 regulates cancer initiation, progression, and metastasis. A previous study scoured cancer genomic databases and found that CFP1 has high mutation rates in colon adenocarcinoma, endometrial carcinoma, and prostate cancers, and even a significant co-occurrence of genetic aberration.^9^ However, studies on the role of CFP1 in LUAD are insufficient.

In this study, we demonstrated that CFP1 was a poor prognostic marker for LUAD patients and promoted the malignant behavior of LUAD. CFP1 deficiency selectively repressed H3K4me3 deposition on the promoter regions of cancer-promoting genes, especially significantly impaired the WNT and TGF-β signaling pathways. These findings highlight the tumor-promoting role of CFP1 in LUAD. The redistribution of H3K4me3 within the genome and subsequent selective activation and inhibition of multiple target genes induced by CFP1 is the implementation way.

## Results

### CFP1 expression is upregulated in LUAD and associated with poor prognosis

**Fig. 1:**
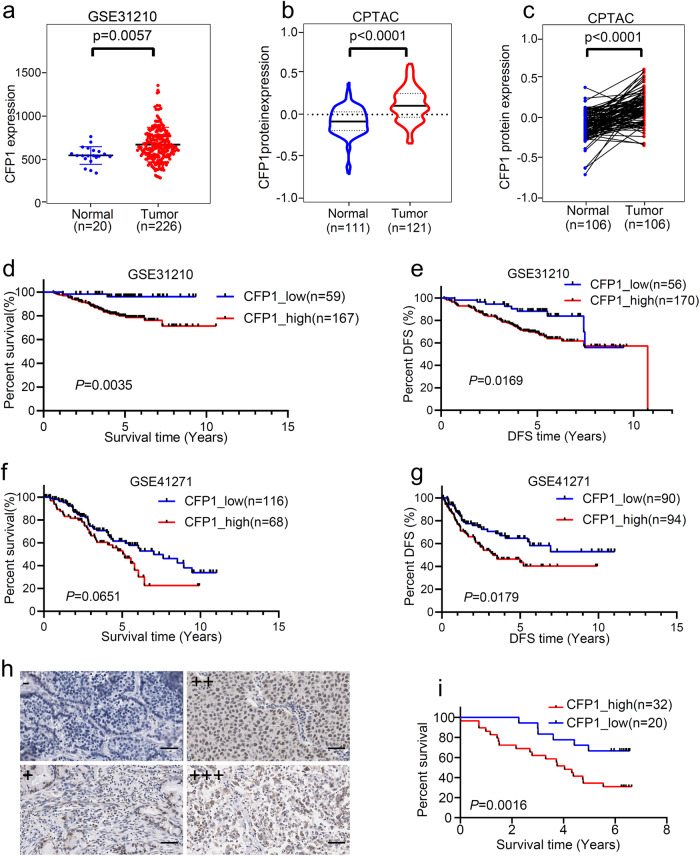
The expression signature of CFP1 in LUAD patient cohorts. **a** Comparison of CFP1 expression levels in tumor tissues and normal tissues from the GSE31210 dataset. Each dot represents a sample. **b** Comparison of CFP1 protein expression from the CPTAC dataset. **c** The paired comparison of CFP1 expression in pairs of tumor and normal tissues from the CPTAC dataset. **d**–**g** The Kaplan–Meier plot represented the OS and DFS of patients stratified by CFP1 expression level. RNA-seq and survival data were obtained from GSE31210 and GSE41271 datasets. **h** The representative immunohistochemistry (IHC) staining image of CFP1 expression in tumor tissues. Samples were obtained in the in-house LUAD cohort (*n* = 52). Scale bars:20 μm. **i** The Kaplan–Meier plot for patients stratified by CFP1 expression level from the cohort in (**h**)

Previous studies have reported that CFP1 expression was a prognostic marker of gastric cancer and that CFP1 could promote ovarian cancer cell proliferation,^17^ so we set out to explore whether there were different CFP1 expressions in LUAD tumor and normal tissues and the prognostic predictive value of CFP1 expression for LUAD patients. We collected transcriptome or proteome data of patients from the datasets GSE31210, and the LUAD cohorts enrolled by TCGA and CPTAC databases, and found that CFP1 expression was significantly upregulated in tumor tissues (Fig. 1a, b, Supplementary Fig. 1a). Paired comparison of the CFP1 protein levels of patients from the CPTAC database and GSE63459 also showed that tumor tissues had higher CFP1 protein expression than normal tissues (Fig. 1c, Supplementary Fig. 1b). To explore whether high CFP1 expression was associated with patients’ clinical outcomes, we conducted survival analyses using GSE31210, GSE41271, and GSE30219 datasets, and the results showed that patients with high CFP1 expression of tumor tissues had shorter overall survival (OS) and disease-free survival (DFS) than patients with low CFP1 expression (Fig. 1d–g, Supplementary Fig. 1c, d). The univariate regression analysis of the GSE30219 dataset displayed that high CFP1 expression was a poor prognosis factor for LUAD patients (Supplementary Fig. 1e). We also included an in-house LUAD patient cohort (*n* = 52) registered in the National Cancer Center and performed immunohistochemical (IHC) scores of patients’ paraffin samples to evaluate CFP1 expression (Fig. 1h), and the survival analysis showed that patients with higher CFP1 expression had worse OS (Fig. 1i). These findings indicated that CFP1 was upregulated in LUAD, and its high expression was a poor prognosis factor.

### CFP1 knockdown suppresses the malignant phenotype of LUAD in vitro and in vivo

**Fig. 2:**
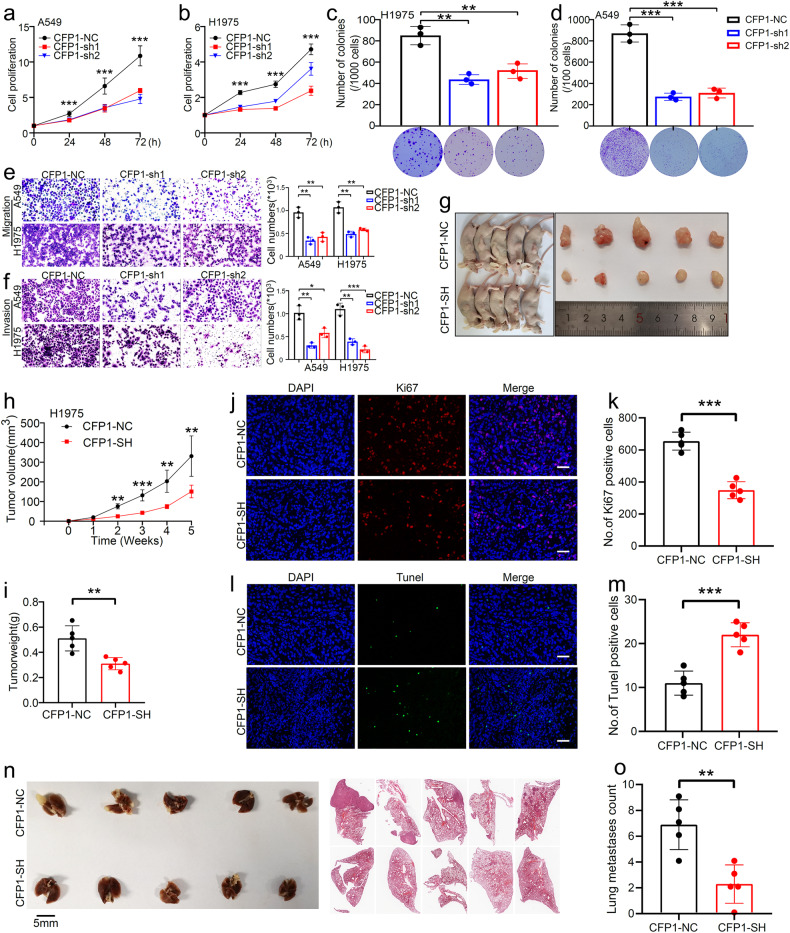
CFP1 knockdown inhibited malignant behaviors of LUAD in vitro and vivo. **a**, **b** The cell proliferation assay using CCK8 for CFP1-knockdown and control cells, including A549 and H1975 cell lines. **c**, **d** The representative colony formation images and quantitative comparison of colony numbers for CFP1-knockdown and control cells, including H1975 and A549 cell lines. (*n* = 3; mean ± SD). **e**, **f** The representative migration and invasion images and quantitative comparison of cell numbers for CFP1-knockdown and control cells. (*n* = 3; mean ± SD). **g** Comparison of subcutaneous tumors in two mice groups. **h** The growth curve of mice subcutaneous tumors in (**g**). **i** Box plot of tumor weights in the two mice groups. **j**, **k** The represent image and statistical bar plot for Ki67 staining of tumor tissues in (**g**). Scale bars:50 μm. **l**, **m** The represent image and statistical bar plot for Tunel staining to detect the apoptosis in tumor tissues. Scale bars:50 μm. **n** Comparison of mice lung metastatic lesions and the microscopic images of hematoxylin-eosin staining sections of lung tissues. **o** The statistical bar plots for the number of metastatic lesions. (for **g**–**o**, *n* = 5; mean ± SD) *P < 0.05; **P < 0.01; ***P < 0.001

Given the correlation between high CFP1 expression and patients’ poor prognosis, we scrutinized whether CFP1 regulated the malignant behavior of LUAD cancer cells. We transfected two human LUAD cell lines A549 and H1975 with shRNA sequences to knockdown CFP1, and the CFP1 knockdown efficiency was detected by western blotting (Supplementary Fig. 2a, b). The cell proliferation and colony formation assays showed that CFP1 knockdown remarkably repressed cell proliferation (Fig. 2a–d). Transwell assay and wound-healing assay demonstrated that cell migration and invasion abilities were remarkably repressed upon CFP1 loss (Fig. 2e, f, Supplementary Fig. 3a–c). The lower sum of S and G2 phase cells while the higher proportion of cells in the G1 phase suggested CFP1 knockdown was likely to interrupt cell cycle (Supplementary Fig. 4d, e). Moreover, loss of CFP1 enhanced early and late apoptosis of cells (Supplementary Fig. 4f, g).

To further validate the cancer-promoting effects of CFP1 on LUAD in vivo, we inoculated CFP1-knockdown and negative control H1975 cells subcutaneously to BALB/c nude mice (*n* = 5 per group). CFP1 ablation markedly diminished tumor growth (Fig. 2g–i). To clarify the mechanism by which CFP knockdown led to tumor growth inhibition, we performed immunofluorescence assays on tumor tissues to detect cell proliferation and apoptosis. The results showed that Ki67 expression was significantly reduced in tumors upon CFP1 deficiency (Fig. 2j, k), along with enhanced cancer cell apoptosis (Fig. 2l, m). Intriguingly, we also found fewer formations of lung metastatic lesions in mice injected with CFP1-knockdown cells through tail veins, implying the potential ability of CFP1 to facilitate tumor metastasis (Fig. 2n, o).

### Loss of CFP1 inhibits caner-promoting pathways while upregulating cancer-repressed pathways

**Fig. 3:**
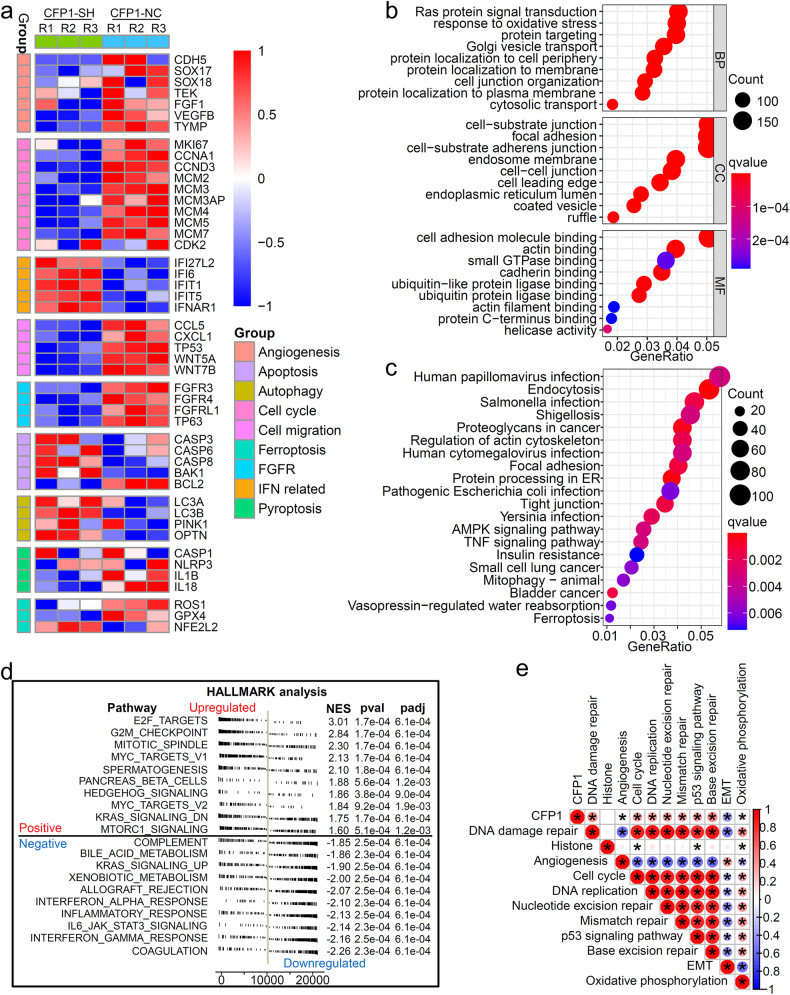
Low expression of CFP1 downregulated oncogenic pathways. **a** Heatmap for gene expression signatures of different cancer-related pathways. The gene expression data was obtained from RNA-seq analyses of the CFP1 knockdown or control A549 cell lines respectively. **b**, **c** The top 9 GO terms (**b**) and top 20 KEGG pathways (**c**) of DEGs between CFP1 knockdown and control cells. DEGs differentially expressed genes, BP biological process, CC cellular component, MF molecular function. **d** The GSEA HALLMARK analysis for CFP1 high expression group and CFP1 low expression group in the GSE30219 cohort. (*n* = 244 for CFP1 low group, *n* = 49 for CFP1 high group. The optimal cut-off value was selected as the patient classification criteria). **e** The correlation coefficient between CFP1 expression level and the enrichment score of different signaling pathways calculated by GSVA analysis for patients from GSE30219. (Red indicates positive correlation; Blue represents negative correlation). GSVA gene set variation analysis, EMT epithelial-mesenchymal transition. *P < 0.05

To study the resulting gene transcriptional alternations which could present the mechanisms behind CFP1 deletion-induced transformation in the malignant phenotype of cancer cells, we performed transcriptome sequencing (RNA-seq) using CFP1-knockdown and negative control A549 cells. The result showed that genes cataloged in oncogenic addiction-related pathways were downregulated in CFP1-knockdown cells, including angiogenesis, cell cycle, and cell migration (Fig. 3a). By contrast, genes constituting cancer-repressing and cell death pathways were upregulated after CFP1 deletion, such as apoptosis, autophagy, and interferon (IFN)-related genes (Fig. 3a). We screened the differentially expressed genes (DEGs) of CFP1-knockdown versus control cells, and the Gene Ontology (GO) and Kyoto Encyclopedia of Genes and Genomes (KEGG) analyses showed that DEGs were primarily enriched to multiple cancer initiation and progression pathways, including Ras protein signal transduction, cell adhesion, AMPK and TNF signaling pathways (Fig. 3b, c). Furthermore, GSEA analysis validated the engagement of CFP1 in tumorigenesis pathways including NF-κB-inducing kinase activity, NOTCH, and TGF-β signaling pathways (Supplementary Fig. 4a, Supplementary Table 2). The GSVA analysis corroborated the significant difference in the activity of various tumorigenic pathways between CFP1-knockdown and control cells, such as the repressed WNT signaling pathway after CFP1 loss even if not statistically significant (Supplementary Fig. 4b, Supplementary Table 3).

To validate our findings in the cancer cell line, we analyzed the transcriptional data of the GSE30219 cohort. Based on the optimal cut-off value defined in Supplementary Fig. 1c, patients were divided into CFP1-high and CFP1-low groups. Supplementary Fig. 4c showed DEGs between two patient groups. Consistent with the enrichment analysis of cell line transcriptome data, the GSEA HALLMARK and GSVA analyses indicated that patient samples with high CFP1 expression were linked to cancer-associated pathways, such as cell proliferation, DNA replication, MYC-, KRAS-, mTOR-targeted signaling, DNA damage repair, and p53 signaling pathway (Fig. 3d, e). Collectively, we proposed that CFP1 ablation extensively influenced gene expression and pathway activation that were associated with malignant progression.

### CFP1 knockdown promotes H3K4me3 modification

**Fig. 4:**
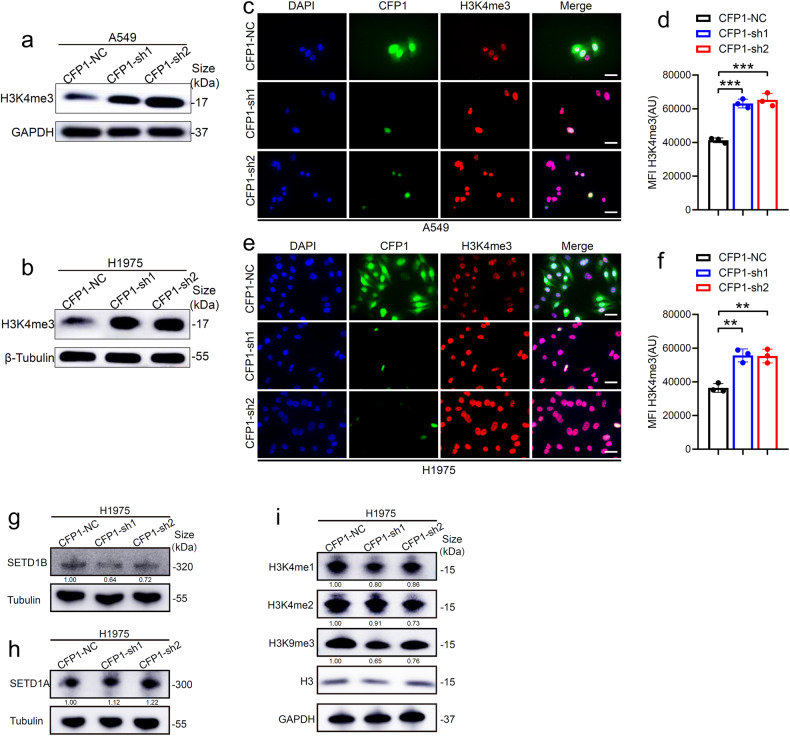
CFP1 knockdown upregulated H3K4me3 modification. **a**, **b** The protein level of total H3K4me3 in CFP1-knockdown or negative control cell lines. **c**, **d** The representative immunofluorescence images for CFP1 and H3K4me3 location and quantitative comparison of immunofluorescence intensity for H3K4me3 in CFP1-knockdown or negative control A549 cell lines. **e**, **f** The representative immunofluorescence images for CFP1 and H3K4me3 location and quantitative comparison of immunofluorescence intensity for H3K4me3 in CFP1-knockdown or negative control H1975 cell lines. (for **c**–**f**, *n* = 3; mean ± SD; Scale bars:10 μm). **g**, **h** The protein level of SETD1A or SETD1B in CFP1 knockdown and negative control cells. **i** The protein level of H3K4me1, H3K4me2, H3K9me3, and histone H3 in CFP1 knockdown and negative control cells. The numbers below the bands represent the relative gray values of corresponding proteins compared to negative control cells

Considering that the primary function of CFP1 is assisting H3K4me3 modification, we assumed that the cancer-restraining effects and transcriptional alternation after CFP1 deficiency were attributed to H3K4me3 modification reprogramming. The whole H3K4me3 protein level in cells was upregulated after CFP1 knockdown, contrary to the methylation function of COMPASS (Fig. 4a, b). Immunofluorescence co-localization displayed that CFP1 localized in the cytoplasm and cell nucleus, and after CFP1 knockdown, the fluorescence intensity of H3K4me3 was visibly enhanced in the cell nucleus (Fig. 4c–f). Our previous studies had reported that high H3K4me3 level in tumor tissues was positively correlated with favorable LUAD prognosis of patients.^18,19^ The antithetical effects of CFP1 and H3K4me3 on patients’ prognosis strongly suggested high CFP1 expression promoted malignancy progression by regulating H3K4me3 modification. SETD1A and SETD1B are the catalytic components of COMPASS, and we speculated whether CFP1 loss affected their expression thereby enhancing H3K4me3 formation. We found that the protein level of SETD1B was reduced upon CFP1 knockdown compared with control cells (Fig. 4g), while SETD1A was slightly upregulated (Fig. 4h). H3K4me1, H3K4me2, and a repressive histone mark H3K9me3 protein level were reduced in cells with CFP1 deficiency (Fig. 4i). These findings implied that CFP1 depletion was likely to promote H3K4me3 formation via the shift of H3K4 methylation from H3K4me1/2 to H3K4me3 without significant impacts SET1 expression, and also engaged in regulating other histone modifications.

### CFP1 regulates oncogenic transcription by establishing genomic H3K4me3 modification

**Fig. 5:**
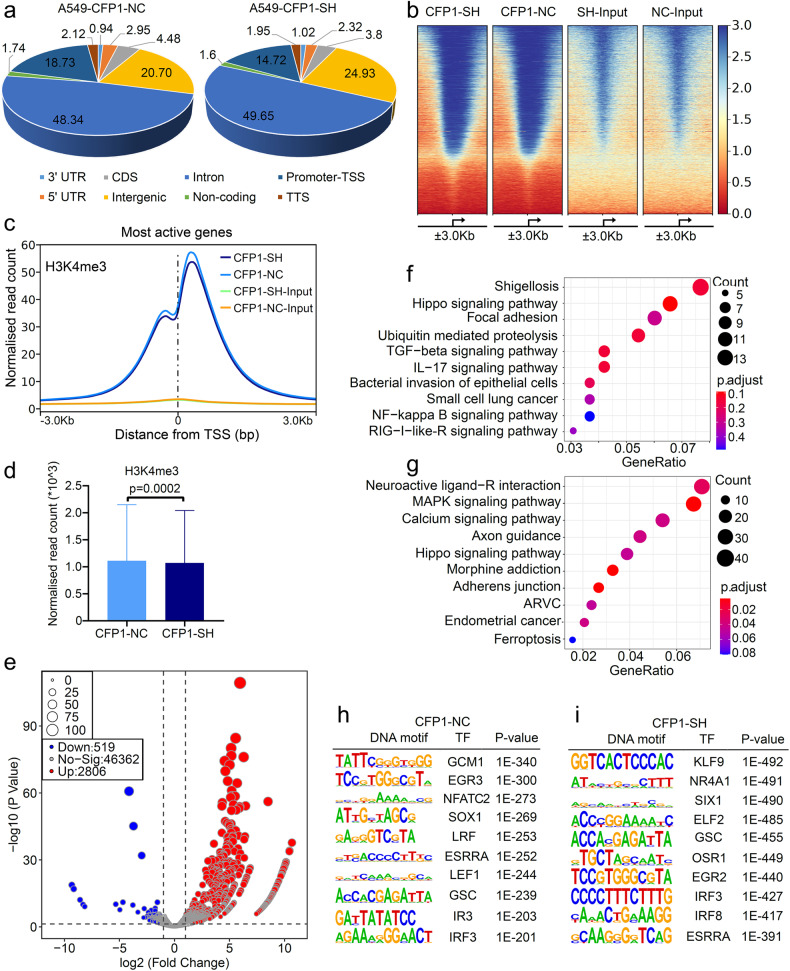
CFP1 regulated selective H3K4me3 deposition within the genome. **a** The pie charts for the distribution of H3K4me3 peaks across the whole genome in CFP1-knockdown and negative control cells. **b** Heatmap for H3K4me3 peaks in CFP1-knockdown and control cells. The profile is centered on TSS, extending upstream 3 kb and downstream 3 kb. **c** Metaplots comparing the accumulation signals of H3K4me3 peaks in negative control or CFP1 knockdown A549 cells. The plot is centered on TSS ( ± 3.0 kb). **d** Box plot for H3K4me3 read counts around TSS ( ± 3.0 kb) in CFP1-knockdown or control cells. **e** The volcano plot for genes that have significant differences in H3K4me3 enrichments within their promoter regions in CFP1-knockdown versus control cells. **f**, **g** The GO and KEGG analysis for DEGs screened in (**e**). **h**, **i** The motif analysis for genomic regions with enriched H3K4me3 peaks in CFP1 knockdown cells or negative control cells

To explain the discordant phenomenon that CFP1 was one indispensable subunit for the H3K4me3 modifying functions of COMPASS whereas its deficiency upregulated the total H3K4me3 expression and significantly repressed the cancer-associated gene expression, we speculated that CFP1 reshaped the H3K4me3 modification signature within the whole genome to induce selective gene activation or inhibition. To this end, we performed chromatin immunoprecipitation sequencing (ChIP-seq) to detect the genome-wide distribution profile of H3K4me3 in CFP1-knockdown and control cell lines (Supplementary Fig. 5a). CFP1 depletion increased the total H3K4me3 peaks compared with negative control (Supplementary Fig. 5b), while reduced H3K4me3 occupancy within promoter-TSS and increased H3K4me3 enrichment within Intron and Intergenic regions (Fig. 5a). Consistently, H3K4me3 enrichment was reduced within gene TSS regions ( ± 3.0 kb) (Fig. 5b–d), suggesting that CFP1 loss led to the reestablishment of genomic modification signature of H3K4me3. We further compared the significance of differences in genes with changed H3K4me3 deposition before and after CFP1 knockdown and surprisingly found that many gene loci with increased H3K4me3 occupancy after CFP1 knockdown had statistical significance, whereas there was no significant difference in most genes with reduced H3K4me3 modification, despite the normalized read count was lower (Fig. 5e). We conducted enrichment analysis to identify the biological functions of these H3K4me3 differentially modified genes and found that they were mostly associated with oncogenic signalings, such as Hippo, TGF-β, MAPK, NF-κB signaling pathways, as well as cancer immunity regulatory pathways IL-17 signaling and the innate immunity pathway RIG-I like-R (Fig. 5f, g). The enriched pathways at least partially were the same targets to the GSEA analysis result using the RNA-seq data of CFP-knockdown or negative control cells, including TGF-β and innate immunity signalings (Supplementary Fig. 4a, b). The overlapping biological pathways found in RNA-seq and ChIP-seq implied that loss of CFP1 triggered epigenetic programming on specific gene loci, and this effect might mediate transcriptional activation. We further performed motif analysis for these H3K4me3-modified chromatin sites (Fig. 5h-i), and the binding sequences of KLF9 and NR4A1 were the top motifs covered with H3K4me3 in CFP1-knockdown cells (Fig. 5i). The pan-cancer analyses demonstrated that the two transcription factors were downregulated in most cancer types, suggesting their potentially inhibitive roles in cancer (Supplementary Fig. 6a, b). Additionally, H3K4me3 was also enriched to the motifs bound by transcription factor families linked to cancer immunity in CFP1-knockdown cells, such as IRF3 and IRF8^20,21^ (Fig. 5i). These findings stressed the genome-wide H3K4me3 modification remodeling ability of CFP1, and in this way, CFP1 deficiency repressed oncogenic transcription while promoting cancer-inhibiting gene expressions.

### CFP1-mediated H3K4me3 deposition at regulated promoters selectively affects tumor-related gene transcription, which mainly leads to abnormal activation of the WNT and TGF-β pathways

**Fig. 6:**
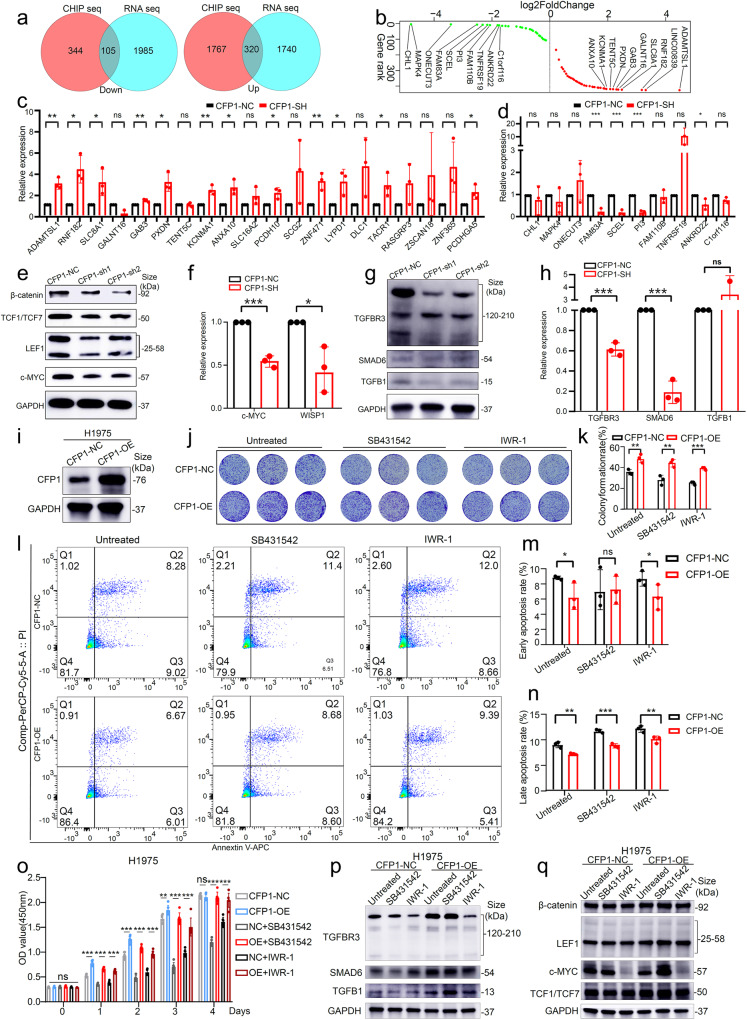
CFP1-mediated H3K4me3 deposition at regulated promoters selectively affected tumor-related gene transcription, which mainly lead to abnormal activation of the Wnt and TGF-β pathways. **a** The Venn plot for overlapping genes with increased/decreased enrichment of H3K4me3 peaks on promoters and increased/decreased transcription levels respectively following CFP1 knockdown in LUAD cells. **b** The rank of genes with the most transcription and H3K4me3 modification changes. **c** The transcriptional level of the top 20 genes with upregulated transcription level and H3K4me3 enrichment in CFP1-knockdown cells compared with control cells by q-PCR. **d** The transcriptional level of the top 10 genes with downregulated transcription level and H3K4me3 enrichment in CFP1-knockdown cells compared with control cells by q-PCR. **e** The protein level of molecules functioning in the WNT signaling pathway. **f** The transcription level of WNT1 inducible signaling pathway protein 1 (WISP1) and WNT-related key molecule c-MYC. **g**, **h** The transcription and protein level of molecules functioning in the TGF-β signaling pathway. (for **c**, **d**, **f**, and **h**, *n* = 3; mean ± SD). **i** The overexpression efficiency of CFP1 in H1975 cell. **j**, **k** The colony formation assay showed the different growth inhibition efficiencies of SB431542 and IWR-1 on control or CFP1-overexpression cells. **l**–**n** The cell apoptosis rates were measured by Annexin-V/PI staining after 24 h of inhibitor treatment (SB431542 50 μM, IWR-1 50 μM). (for **j**–**n**, *n* = 3; mean ± SD). **o** CCK8 assay measured cell proliferation rate at different time points after inhibitor treatment. Six replicates were set for each group, and the statistical difference of OD value between groups was analyzed by Student’s *t*-test. (*n* = 3; mean ± SD). **p** The protein expression level of molecules functioning in TGF-β signaling pathways in H1975 cells upon CFP1 overexpression along with the two pathway inhibitors treatment. **q** The protein expression level of molecules functioning in WNT signaling pathways in H1975 cells upon CFP1 overexpression along with the two pathway inhibitors treatment. *P < 0.05; **P < 0.01; ***P < 0.001; ns not significant

To identify those genes that were indeed regulated by the epigenetic regulation axis of CFP1/H3K4me3, we selected the genes (*n* = 320) which showed upregulated expression by RNA-seq and simultaneously increased H3K4me3 deposition within their promoter regions following CFP1 deletion (Fig. 6a, Supplementary Table 4). Likewise, genes (*n* = 105) that were downregulated and had less promoter enrichment of H3K4me3 modification were also screened (Fig. 6a, Supplementary Table 5). We performed quantitative PCR (qPCR) to validate the transcriptional changes of these top-rank upregulated and downregulated genes (Fig. 6b–d). The downregulated genes, such as FAM83A, SCHL, and ANKRD22 were previously reported to promote cancer progression,^22–25^ by contrast, the upregulated genes including GAB3,^26^ SLC8A1,^27^ KCNMA1,^28^ and ZNF471,^29^ were identified as cancer-inhibiting genes in various cancer types, and negatively correlated with poor prognosis of patients. To investigate their roles in LUAD, we analyzed the expression and prognostic ability of these genes using the TCGA LUAD dataset. The upregulated genes including GAB3, SLC8A1, KCNMA1, and ZNF471 following CFP1 knockdown, were expressed at low levels in tumor tissues and were associated with favorable prognosis of LUAD patients (supplementary Fig. 7a, c). whereas the downregulated genes induced by CFP1 deficiency, such as FAM83A and ANKRD22, were upregulated in tumor tissues and associated with poor prognosis in the TCGA LUAD dataset (Supplementary Fig. 7b, d).

To further explore the primary oncogenic signaling pathways that were impaired by CFP1 loss, we performed the functional enrichment analysis of the downregulated gene set (*n* = 105) and found that WNT and TGF-β signaling pathways were enriched (Supplementary Table 6). The critical target gene c-MYC, as well as the essential function molecules LEF1, TCF1/TCF7, and β-catenin of the WNT pathway were downregulated upon CFP1 deficiency,^30^ and the transcriptional level of WNT1 inducible signaling pathway protein 1 (WISP1) was also significantly reduced (Fig. 6e, f). In addition, we found that the TGF-β pathway-associated genes TGFB1 and TGFBR3 were also downregulated in CFP1-knockdown H1975 cells (Fig. 6g, h). The protein level of SMAD6, an inhibitory factor of the TGF-β pathway,^31^ had no significant change after CFP1 knockdown although SMAD6 transcription decreased in qPCR and RNA-seq (Fig. 6g, h, Supplementary Table 6). The nonsignificant difference in TGFB1 expression showed by qPCR might be due to its minimal transcription level (Fig. 6h).

In an attempt to better illustrate how H3K4me3 modification mediated by CFP1 regulates LUAD progression through the WNT and TGF-β pathways, we constructed the CFP1-overexpression cell line, and as expected ectopic CFP1 expression enhanced the ability of cell proliferation and colony formation, and reduced cell apoptosis rates (Fig. 6i–o), along with the increased expression of β-catenin, LEF1, TCF1/TCF7, TGFBR3, SMAD6, and TGFB1 (Fig. 6p, q). Especially, although the WNT (IWR-1) and TGF-β (SB431542) pathway inhibitors impaired the cancer-promoting effects of CFP1 overexpression, they more intensively inhibited the growth of negative control cells (Fig. 6j–o). This finding suggested that CFP1 overexpression activated WNT and TGF-β signaling pathways and enhanced cell malignant performances, which were not completely blocked by inhibitors. However, except for c-MYC, there were no or only slight changes in other effect molecules, including β-catenin, LEF1, SMAD6, TCF1/TCF7, and TGFB1 after inhibitor treatment (Fig. 6p, q). TGFBR3 expression was also markedly repressed after IWR-1 treatment, this unexpected finding implied that the WNT pathway might interactively modulate the TGF-β signaling pathway (Fig. 6p). Taken together, the CFP1-mediated genomic H3K4me3 modification reshaping was implicated in selectively activating cancer-associated gene expressions, and the WNT and TGF-β signaling pathways played dominant roles in contributing to cancer-promoting effects mediated by CFP1.

## Discussion

This study reveals that CFP1 plays a cancer-promoting role in LUAD, and high CFP1 expression in tumor tissues is a poor prognostic factor. Mechanistically, the CFP1-mediated establishment of genomic H3K4me3 modification profile activates various oncogenic gene expressions and enables the initiation of corresponding signaling pathways, especially the WNT and TGF-β pathways, which predominantly contribute to the malignant progression of LUAD.

As the fundamental element of the epigenetic regulatory network, H3K4me3 modification extensively participates in regulating cancer-related gene expressions.^32^ We identified that CFP1, the non-catalytic subunit of COMPASS, was highly expressed in LUAD tissues and promoted cancer cell proliferation, migration, and invasion while inhibiting cell apoptosis. ChIP-seq combined with RNA-seq showed that CFP1 ablation selectively alternated genome-wide H3K4me3 deposition and played a dual effect on the expression of genes implicated in cancer promotion or suppression. Notably, we found that the deletion of CFP1 increased the overall level of H3K4me3 in LUAD cell lines, which was inconsistent with its known function in assisting histone methylation. Different studies on how CFP1 affects the catalytic methylation ability of the SET1/COMPASS complex provided two contradictory conclusions. Lee et al. found that the mass of the Set1 complex was reduced in murine embryonic stem cells lacking CFP1, and these cells carried elevated three methylated H3K4 modification levels and reduced levels of heterochromatin. Thus, the authors thought that CFP1 inhibited the activity of the Set1/CFP1 complex.^33^ In vertebrate animals, most promoters employ unmethylated CGIs.^34^ Previous studies disclosed that CFP1 recognized these nonmethylated CG dinucleotides and recruited the SETD1A methyltransferase complex to catalyze H3K4 trimethylation within these regions.^35–37^ Therefore, researchers proposed that the deletion of CFP1 led to decreased H3K4me3 levels of specific genes and global inactivated transcription, thereby inducing several cellular dysfunctions or aberrant transcription events.^38–41^ In addition, a recent study found that CFP1 only slightly activated COMPASS methyltransferase activity in vitro.^42^ So the mechanisms underpinning the stimulatory or inhibitory effects of CFP1 on the Set1 complex are poorly understood. Our study provides a novel perspective that CFP1 can selectively regulate H3K4me3 deposition within the genome in LUAD and participates in tumorigenesis. A study corroborating our view reported that ablation of CFP1 has distinct effects on H3K4me3 formation at different gene loci: H3K4me3 positing in CGI-related genes would suffer drastic loss after CFP1 ablation, while ectopic H3K4me3 peaks would appear at many other regulatory regions. This finding implied that CFP1 elimination triggered H3K4me3 “leakage”.^14^ Notably, recent studies expand the originally defined knowledge of CFP1 genomic occupation pattern and transcriptional output regulation mediated by CFP1. Instead of being restricted to active CGIs, CFP1 also occupies non-CGI TSSs and enhancers of transcribed genes in two human hematopoietic cell types.^43^ The facilitating effects of CFP1 to H3K4me3 formation are more tightly associated with gene specificity rather than transcription output levels.^39^

In our study, WNT and TGF-β signaling pathways were identified as the predominant downstream oncogenic pathways regulated by epigenetic regulation CFP1/H3K4me3 axis. CFP1 upregulated expression of β-catenin, LEF1, TCF1/TCF7, c-MYC, TGFBR3, and TGFB1, and the cancer-promoting effects of CFP1 were not completely repressed by pathway inhibitors. Previous studies had found SETD1A maintained the WNT pathway activation by stabilizing β-catenin,^44^ and H3K4 trimethylation induced by demethylase inhibitor blocked the TGF-β-induced invasion and migration of cancer cells, suggesting H3K4me3 modification was one of the essential epistatic regulators of the two cancer driver pathways.^45^ Our study supplemented that the non-catalytic composition of histone modification enzymes could reshape the histone mark signature of the genome. However, the specific molecular events by which CFP1 regulates histone methylation are unclear.

CFP1 is also a promising novel target for developing the new generation DNMT inhibitor based on its functional interaction with DNMT, and the efficiency of DNMT1/CFP1 complex disruption has been displayed in enhancing chemotherapy sensitivity in glioblastoma.^46^ Given these findings, we propose that CFP1 is a critical epigenetic regulator for LUAD progression and might be a novel epigenetic therapeutic target. Nevertheless, how CFP1 regulates H3K4me3 modification and cancer-associated pathways in different cancers remains to be investigated.

Collectively, the study sheds light on the genome-wide regulation effects of CFP1 on H3K4me3 modification in LUAD cells, in this way, CFP1 selectively enhances cancer-promoting transcription and inhibits cancer-inhibiting transcription. CFP1 is a novel epigenetic target and prognostic factor for LUAD.

## Materials and methods

### Dataset source and preprocessing

Six independent public LUAD cohorts with proteomics or genomics data and clinical information were used in our studies. 497 LUAD tissues and 54 normal controls were obtained from The Cancer Genome Atlas (TCGA) website (https://portal.gdc.cancer.gov/). The RNA-seq and survival data of LUAD patients in GSE31210 (*n* = 246), GSE41271 (*n* = 184), GSE30219 (*n* = 293), and GSE63459 (*n* = 33) were downloaded from Gene Expression Omnibus (GEO) datasets (http://www.ncbi.nlm.nih.gov/geo). A LUAD cohort (*n* = 232) with protein expression data was downloaded from the CPTAC website (https://proteomics.cancer.gov/programs/cptac). We also collected a LUAD patient cohort (*n* = 52) with survival data at the National Cancer Center/National Clinical Research Center for Cancer/Cancer Hospital, Chinese Academy of Medical Sciences, and Peking Union Medical College (Beijing, China). R version 4.0.5 software was used to normalize and process the data.

### Patients, surgical specimens, and immunohistochemistry

The tumor tissues and tumor-adjacent tissues were obtained from 52 patients diagnosed with LUAD and operated on at the Department of Thoracic Surgery of the National Cancer Center from 2011 to 2012, under the approval of the Institutional Review Committee of the Cancer Hospital, Chinese Academy of Medical Sciences. The paraffin-embedded samples of these patients were sectioned and stained by CFP1 antibody (1:1000, ab198977). The immunohistochemical staining (IHC) intensity was recorded as 0 (no staining), 1 (weak), 2 (moderate), and 3 (strong). The score of stain area was recorded as 0 (0–5%), 1 (6-25%), 2 (26-50%), 3 (51-75%), and 4 ( > 75%). The IHC score was calculated as the intensity score multiplied by the stain area score, and two pathologists performed the evaluation independently.^47^

### Survival analysis

The survival analysis for LUAD patients grouped according to CFP1 expression level or H3K4me3 enrichment score was performed with R package “survival” and “survminer”. The value for grouping patients was set as the optimal cut-off value.^48,49^

### Enrichment analysis

The Gene Ontology (GO) and Kyoto Encyclopaedia of Genes and Genomes (KEGG) pathway analyses of the DEGs between CFP1-knockdown and vector-only control cells were performed using R packages “clusterProfiler”, “org.Hs.eg.db”, “enrichplot”, and “ggplot2”. The Gene Set Enrichment Analysis (GSEA) analysis was conducted with GSEA software (V.4.1.0). The Gene Set Variation Analysis (GSVA) of cancer-associated pathways was performed with R package “gsva”, and the enrichment scores of pathways were calculated based on RNA-seq data.

### Cell culture

The lung adenocarcinoma cell lines H1975 and A549 were purchased from American Type Culture Collection (ATCC). Cells were cultured in medium RPMI 1640 (Gibco) supplemented with 10% fetal bovine serum (FBS, Gibco), 100 U/mL penicillin, and 100 μg/mL streptomycin (Gibco) at 37 °C, 5% CO_2_ in the incubator.

### Cell transfection

A set of two different plasmids (Genechem) carrying shRNA (5' CGACTCTTCTGTGATGTGTAT 3' and 5' CATCCGGATCACTGAGAAGAT 3') targeting CFP1 were inserted in lentiviral vectors respectively. Virus particles were produced by co-transfected 293 T cells with target plasmids and virus package plasmids (Syngentech) according to the manufactory protocol of the Lipofectamine 3000 kit (Invitrogen). After 48 h, viral supernatant was collected and transduced to target cells with 8 μg/mL polybrene (Beyotime). Puromycin (Beyotime) was diluted in the complete culture medium (final concentration 2 μg/mL) to select transfected successfully cells for 2 weeks. The CFP1 overexpression plasmid and vector control plasmid (Genechem) were transfected in H1975 cells with Lipofectamine 3000 (Invitrogen). After 48 h, the protein overexpression efficiency was detected by western blotting.

### Cell proliferation assay

CCK-8 assay (DOJINDO) was performed to assess cell proliferation. CFP1-knockdown or control A549 cells (1 × 10^3^ cells per well) were plated in 96 well plates for preincubation. Before each CCK8 test, the old medium was removed and 90 μL culture medium supplemented with 10 μL CCK-8 was added to each well, and plates were incubated in the incubator (37 °C, 5%CO_2_) for 2 h. The OD values were measured at 450 nm with a microplate reader. The same treatment was applied to CFP1-knockdown or control H1975 cells. In the WNT and TGF-β pathway inhibitors treatment assay, CFP1-overexpression or control H1975 cells were plated in 96 well plates (1 × 10^3^ cells per well). SB431542 (50 μM, Selleck) and IWR-1 (50 μM, Selleck) were diluted in culture medium and added to plates. CCK8 assay was performed at 0, 24, 48, 72, and 96 h respectively. Differences in OD value were analyzed by Student’s t-test at different time points.

### Western blotting

During protein extraction, cells were collected and lysed by RIPA lysis buffer (Servicebio) with the addition of proteinase inhibitor cocktail (APPLYGEN) on ice for 15 min. The supernatants were stored at −80 °C after centrifugation. The concentrations of total proteins were measured using a BCA protein assay (ThermoFisher Scientific), and 10 μg proteins of each sample were used for western blotting. Proteins were separated by SDS-PAGE gel electrophoresis and transferred onto PVDF membranes (Millipore). Following blocked with 5% BSA solution for 1 h, the membranes were incubated at 4 °C overnight with primary antibodies to CFP1 (1:2000, ab198977), H3K4me3 (1:1000, CST #9751), GAPDH (1:1000, CST#2118), SETD1A (1:5000, Proteintech), SETD1B (1:1000, ab300479), H3K4me1 (1:5000, ab176877), H3K4me2 (1:2000, ab32356), H3K9me3 (1:1000, ab176916), Histone H3 (1:5000, Proteintech), TGFBR3 (1:500, sc-74511), TGFB1 (1:500, sc-130348), SMAD6 (1:500, sc-25321), TCF1/TCF7 (1:1000, CST#2203), LEF1 (1:1000, CST#2230), c-MYC (1:1000, CST#18583), β-Catenin (1:1000, CST#8480), and Tubulin (1:1000, ab7921). Then membranes were incubated with HRP-conjugated anti-rabbit or anti-mouse secondary antibody (1:3000, CST#7074 or CST#7076) for 2 h at room temperature, and the protein bands were detected with the chemiluminescence detection kit (Immobilon™ Western Chemiluminescent HRP Substrate) and imager.

### Wound-healing assay and transwell assay

For the wound-healing assay, cells were cultured in 6-well plates. After cells reaching to 90% confluence, a sterile pipette tip was used to generate a clean scratch across the center of the cell layer. Photographs were taken at 0 h, 24 h, and 48 h under the microscope. The cell migration distance was estimated by Image J software. For transwell assay, 7.5 × 10^5^ cells were seeded onto the upper chamber coated with Matrigel (invasion assay) or without Matrigel (migration assay) in the culture medium without FBS, and 600 μL culture medium containing 10%FBS was added to the bottom chamber. The transwell installation was incubated at 37 °C with 5% CO_2_ for 48 h. Then the chambers were fixed by methanal followed by invasive and migratory cells on the lower surface were stained with 0.1% crystal violet (Beyotime) and counted under the microscope.

### Cell cycle assay

Cell cycle detection was performed according to the kit’s protocol (KGA511, KeyGEN BioTECH). Briefly, cells were collected, washed, and fixed with 70% cold ethanol for 3 h at 4 °C. Cells were washed to remove fix solution followed by staining with 500 μL PI/RNase A working solution (PI: RNase A = 9:1) at room temperature in the dark for 60 min. The cell numbers in G1, S, and G2 phases were collected on LSR II Flow Cytometer and systems (BD Biosciences) and analyzed with FlowJo (version 10.0.7).

### Apoptosis assay

Cell apoptosis was measured by Annexin V/propidium iodide (PI) staining according to the kit protocol (AT107, MULTISCIENCES). Cells were collected, centrifuged, washed for one time, and resuspended in 500 μL binding buffer. Then 5 μL Annexin V-APC and 10 μL PI were added to each tube. After 15 min of incubation in the dark, the apoptosis cell numbers were collected on LSR II Flow Cytometer and systems (BD Biosciences) and analyzed with FlowJo (version 10.0.7). In the inhibitors treatment assay, cell apoptosis detection was conducted after 24 h of treatment.

### Colony formation assay

H1975 cells were seeded uniformly in six-well plates at the density of 1000 cells/well. SB431542 (50 μM) and IWR-1(50 μM) were diluted in complete medium and added to the treatment groups for 10 days of incubation at 37 °C, 5% CO_2_. The control groups were treated with DMSO. When the number of cells was >50 in one cell colony, cells were washed, fixed by 4% paraformaldehyde, and stained by crystal violet (Beyotime). The colony formation rate of one well was calculated by: (staining area/total area) ×100%. The area was measured by ImageJ.

### Real-time quantitative PCR

1 × 10^6^ cells were collected to extract RNA using the RNA-Quick Purification Kit (RN001, ES Science) following the manufacturer’s instruction. Then 1 μg total RNA was reverse transcripted using the Reverse Transcription Kit (AU341, TransGen) according to the manufacturer’s protocol, and quantitative PCR was performed with Primers (Generay) and SYBR Green dye (AQ601, TransGen). Comparison for gene expression was calculated using the ΔΔCt method. The primers and oligonucleotides were listed in Supplementary Table 1.

### RNA and ChIP extraction

RNA was extracted using RNA-Quick Purification Kit (RN001, ES Science) following the manufacturer’s instructions, and RNA-seq libraries were constructed using the TruSeq Stranded Total RNA with Ribo-Zero kit (Illumina). For ChIP extraction, cells were plated in 15 cm culture dishes. Cells were collected for subsequent experiments when the culture reached ~70% confluency (~10^6^ cells for each reaction). ChIP extraction was performed using SimpleChIP Enzymatic Chromatin IP Kit (CST#9003) following the manufacturer’s instructions. The primary antibody included H3K4me3 (2 μg antibody for 25 μg chromatin, ab8580). Two biological duplications were performed for the ChIP-seq of each cell line. ChIP-seq libraries were constructed with the KAPA HTP library preparation kit (KAPA Biosystems) and loaded onto an Illumina HiSeq X Ten for sequencing. RNA-seq libraries were sequenced with Illumina HiSeq 2500.

### Sequencing data processing

RNA-seq and ChIP-seq data quality were assessed with FastQC v0.11.3. Adapter sequences and low-quality data were filtered and excluded. Quality reads of RNA-seq and ChIP-seq were mapped to human genome hg19 by TopHat v2.0.9 and Bowtie v2 software respectively, and only uniquely mapped reads were subjected to further analyses. Sam files were converted to Bam files with Samtools v1.8. Bam format files were used for peak calling with MACS v2.1.0 software using default parameters. The input sample was the control and the initial threshold q-value of 0.05 was the cutoff value. The output of the peak files was converted to bigwig files and viewed using IGV software. Meta plots and heatmaps were produced by deeptools. Motif analysis was conducted with Homer v4.1.5. The differential analysis was performed using R package “EdgeR”, and the screening criteria for significantly different genes were *p*-value < 0.05 and |log_2_FC | >1.

### Immunofluorescent staining

The cell coverslips were washed with PBS three times and fixed with 4% paraformaldehyde. Membrane permeation was performed using 0.25% Triton X-100 (diluted in PBST). Cells were incubated in primary antibodies at 4 °C overnight. After removing the primary antibodies, cells were incubated in the secondary antibodies of the same species as the primary antibodies for 1 h in the dark condition. Following nuclear staining by DAPI, the coverslips were sealed and observed under the fluorescence microscope. The primary antibodies include: CFP1 (1:500, ab198977) and H3K4me3 (1:500, ab12209). The secondary antibodies include anti-rabbit IgG (H + L), F(ab’)2 Fragment (Alexa Fluor® 488 Conjugate) (1:1000, CST #4412) and anti-mouse IgG (H + L), F(ab’)2 Fragment (Alexa Fluor® 594 Conjugate) (1:1000, CST #8890).

The Tunel staining of paraffin sections of mouse tumor tissues was conducted according to the kit instruction (Servicebio G1504). Briefly, the sections were dewaxed in xylene, gradient Ethanol solution (100%, 90%, 70%), and distilled water. Sections were covered in Proteinase K (20 μg/mL) solutions and incubated for 30 min at 37 °C. Then 0.5% Triton X-100 was used for permeation. Tunel stain solution was prepared according to the manufacturer’s protocol and added to sections incubating for 60 min at 37 °C. After the DAPI staining, sections were observed with a fluorescence microscope (Nikon eclipse C1).

### In vivo studies

Female BALB/c nude mice (6-week-old) were purchased from Huafukang Bioscience (Beijing, China). Mice were housed in a specific pathogen-free animal facility. 2 × 10^6^ H1975 cells were resuspended in 100 μL PBS and subcutaneously injected into the flanks of mice (*n* = 5 for each group). Mice were euthanized and tumors were stripped until the longest diameter was up to 15 mm. The tumor volume was calculated by: V = *L* × *W*^2^ × 0.5 (*L* = longest diameter and W=shortest diameter). The lung metastasis model was established by injecting tumor cells into mouse tail veins (*n* = 5 for each). Lung tissues were removed after 6 weeks.

### Statistical analysis

Pearson correlation coefficient was calculated in correlation analyses. Two-tailed unpaired Student’s *t*-test was used for comparison between the two groups. Multiple groups were compared using one-way ANOVA. All data were represented as mean ± SD or mean ± SEM. *P*-value < 0.05 was considered statistically significant. R version 4.0.5 and GraphPad Prism v8 were used for data analysis.

### Supplementary information

Supplementary materials

Original western blot results

## Data Availability

Data will be available for download at NCBI databases, https://www.ncbi.nlm.nih.gov/, BioProject accession number PRJNA1004838 for RNA-seq data and PRJNA1004875 for ChIP-seq data. Other data are available in the main text or the supplementary materials.
