# Supplementary material for: CXXC finger protein 1 (CFP1) bridges the reshaping of genomic H3K4me3 signature to the advancement of lung adenocarcinoma
Source: Signal Transduct Target Ther. 2023 Sep 21;8:369. doi: 10.1038/s41392-023-01612-3 (PMC10514036; doi:10.1038/s41392-023-01612-3)
Supplement: Supplementary file 1 — Supplementary materials [file 41392_2023_1612_MOESM1_ESM.docx]

Supplementary Materials for

**CFP1 regulating genomic H3K4me3 signature reshaping links to lung adenocarcinoma progression**

Tao Fan^1^#, Chu Xiao^1^#, Hengchang Liu^2^, Yu Liu^3^, Liyu Wang^1^, He Tian^1^, Chunxiang Li^1^, Jie He^1^

^1^Department of Thoracic Surgery, National Cancer Center/National Clinical Research Center for Cancer/Cancer Hospital, Chinese Academy of Medical Sciences and Peking Union Medical College, Beijing, 100021, China

^2^Department of Colorectal Surgery, National Cancer Center/National Clinical Research Center for Cancer/Cancer Hospital, Chinese Academy of Medical Sciences and Peking Union Medical College, Beijing, China

^3^Department of Intervention, National Cancer Center/National Clinical Research Center for Cancer/Cancer Hospital, Chinese Academy of Medical Sciences and Peking Union Medical College, Beijing, China

#Tao Fan and Chu Xiao contributed equally.

Correspondence should be addressed to Jie He ([prof_jiehe@yeah.net](mailto:prof_jiehe@yeah.net)) and Chunxiang Li ([lichunxiang@cicams.ac.cn](mailto:lichunxiang@cicams.ac.cn));

**This PDF file includes:**

Supplementary figure 1-7

Tables S1 to S6

**Supplementary figure 1**


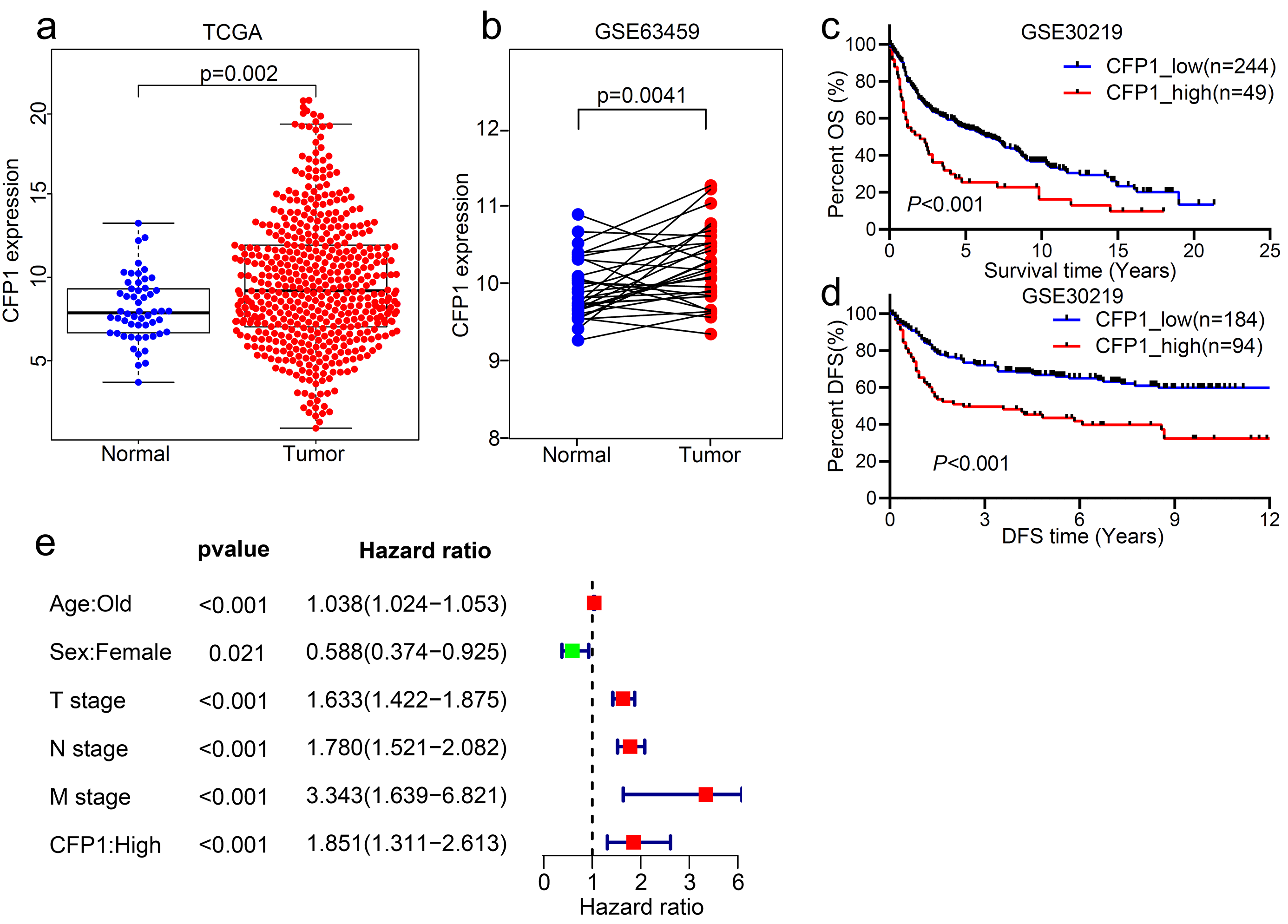


**a** Comparison of CFP1 expression in normal and tumor tissues of LUAD patients from the TCGA dataset.

**b** Comparison of CFP1 expression in normal and tumor tissues of LUAD patients from the GSE63459 dataset.

**c-d** The OS and DFS analysis of patients with high or low CFP1 expression from the GSE30219 dataset. The optimal cut-off value was selected as the patient classification criteria.

**e** The univariate regression analysis of CFP1 expression and other clinical characteristics of patients from the GSE30219 dataset.

**Supplementary figure 2**


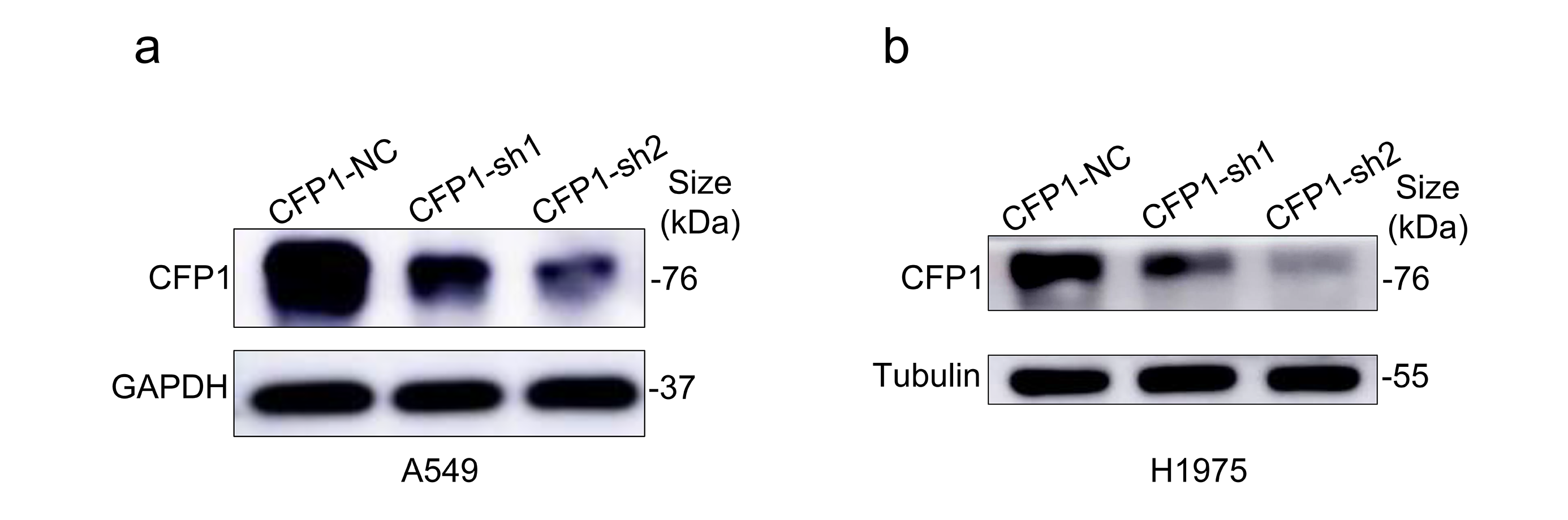


**a-b** Western blotting detecting the knockdown efficacy of CFP1 in A549 and H1975 cell lines.


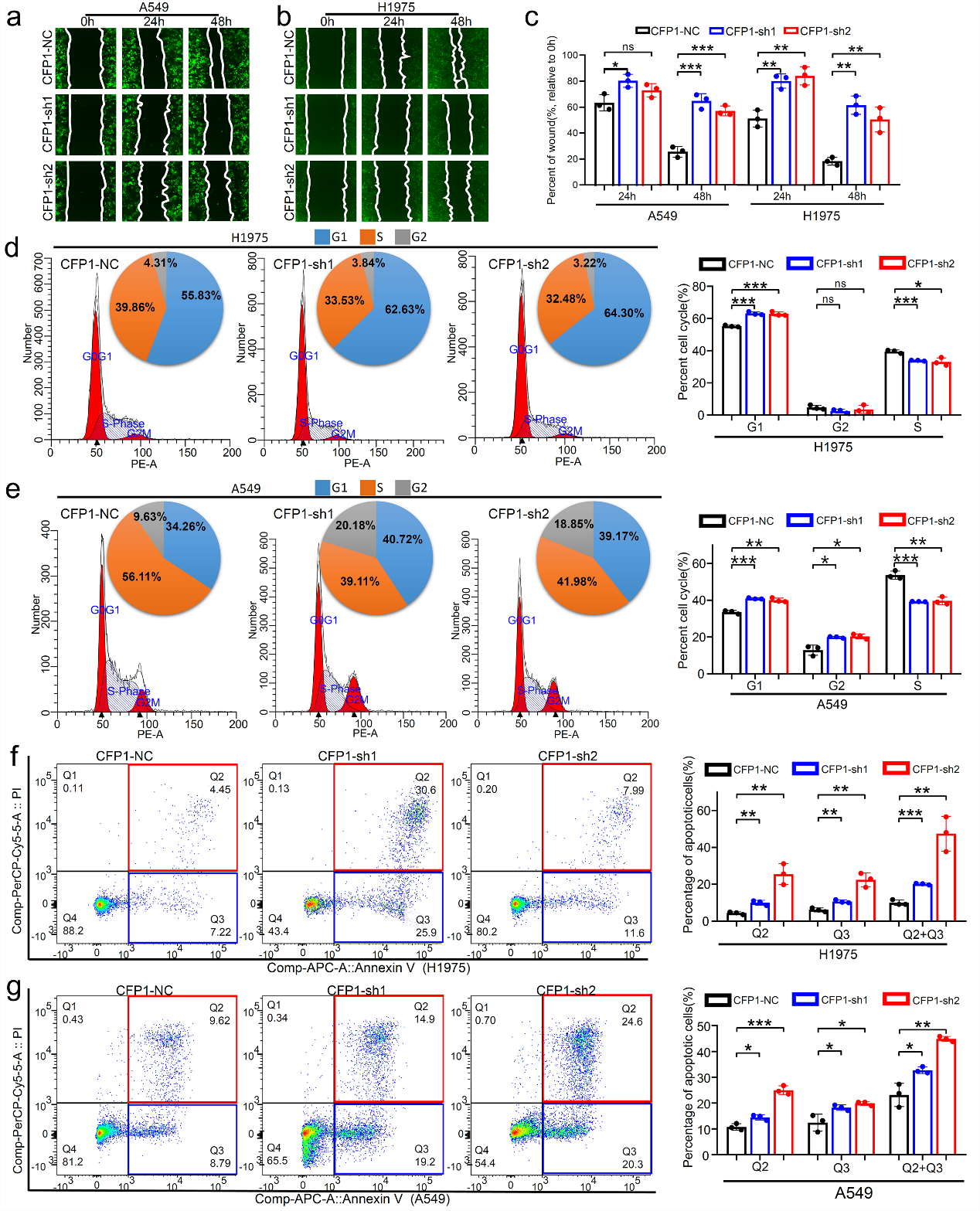


**Supplementary figure 3**

**a-b** Wound healing assay was used to determine cell migration ability in A549 and H1975 cells.

**c** Quantification of the scratch wound areas. Data are expressed as the percentage of the remaining area to the initial scratch area.

**d-e** Flow cytometry was used to detect H1975 and A549 cell cycle.

**f-g** H1975 and A549 cell apoptosis was evaluated by flow cytometry.

(for **a-g**, n=3; mean ± SD). **P*< 0.05; ***P* < 0.01; ****P* < 0.001; ns not significant

**Supplementary figure 4**


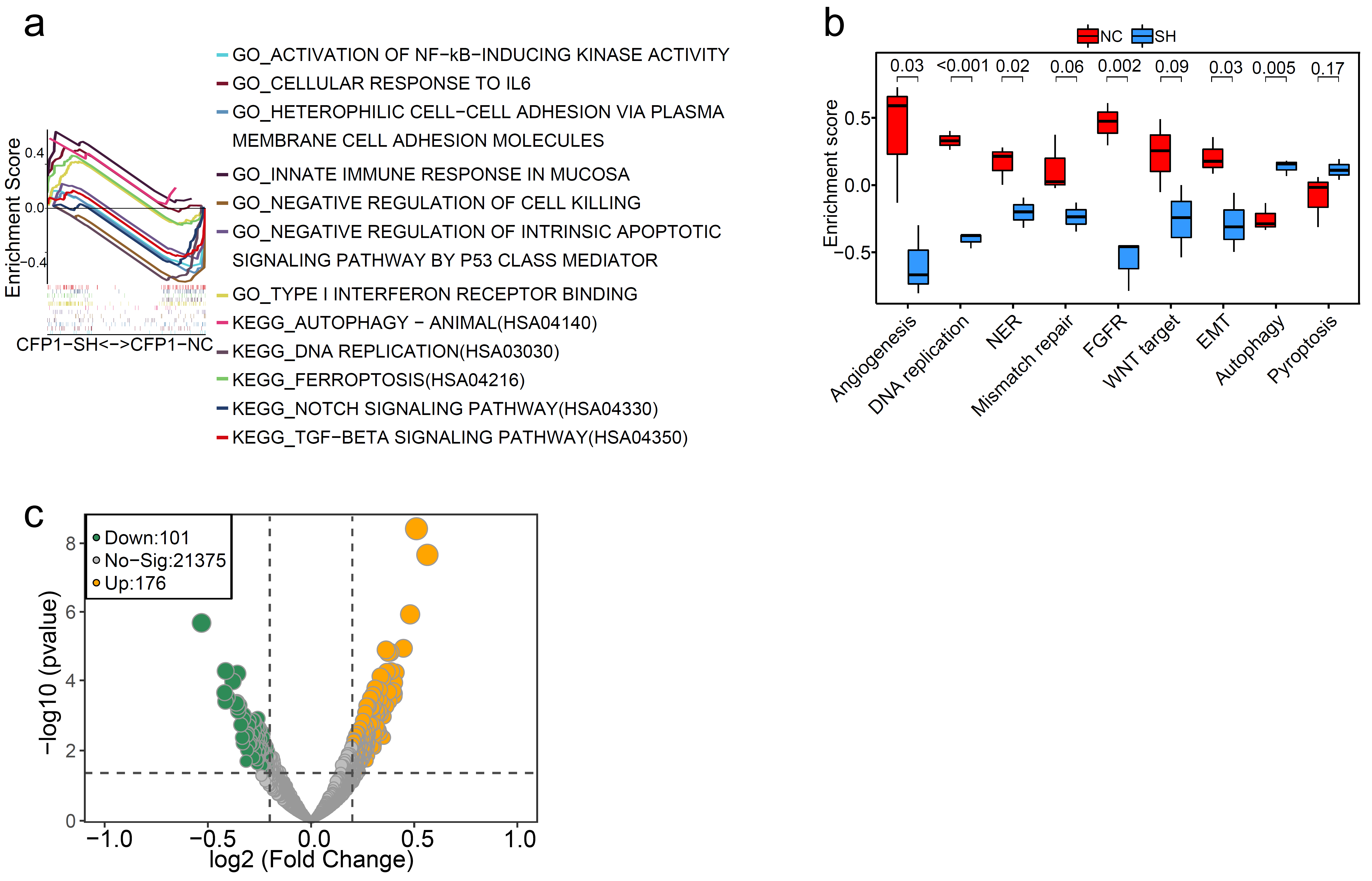


**a** The GSEA analyses of CFP1 knockdown A549 cells and negative control cells.

**b** The GSVA analysis for canonical cancer-associated pathways in CFP1 knockdown and control A549 cells. NER: nucleotide excision repair; EMT: epithelial-mesenchymal transition.

**c** The volcano plots of DEGs between two patient groups classified according to Supplementary figure 1c.

**Supplementary figure 5**


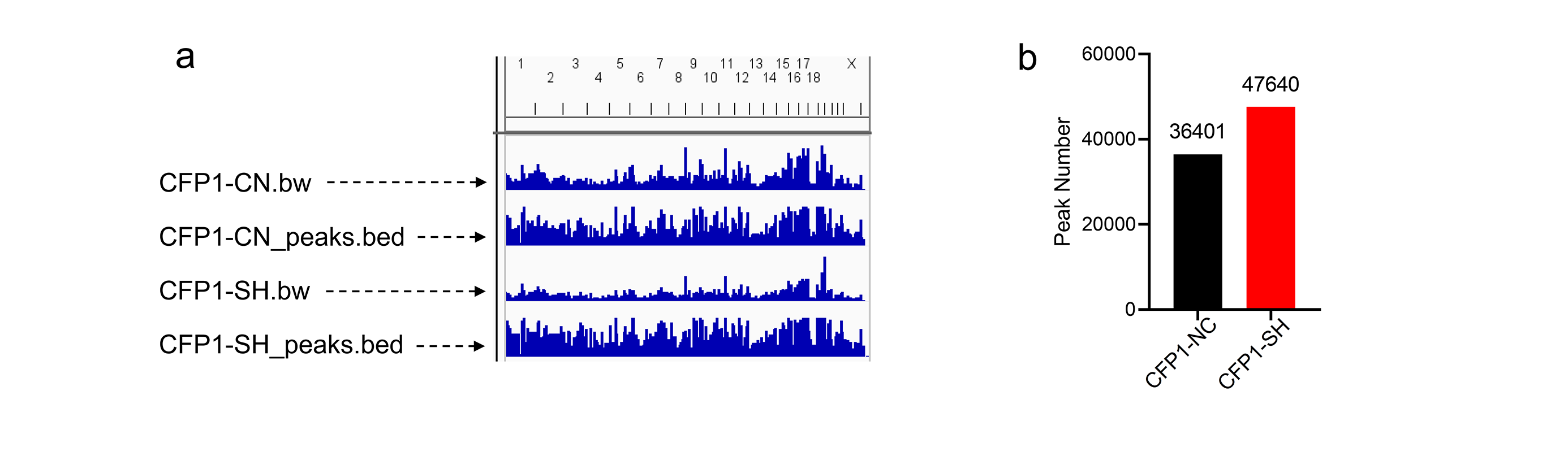


**a** ChIP-seq tracks of H3K4me3 occupancy at whole chromosomes in CFP1-NC and CFP1-SH A549 cells.

**b** H3K4me3 occupancy peak number in CFP1-NC and CFP1-SH A549 cells. Peak calling was performed by MACS2. A q value = 0.05 served as the cutoff criteria.

**Supplementary figure 6**


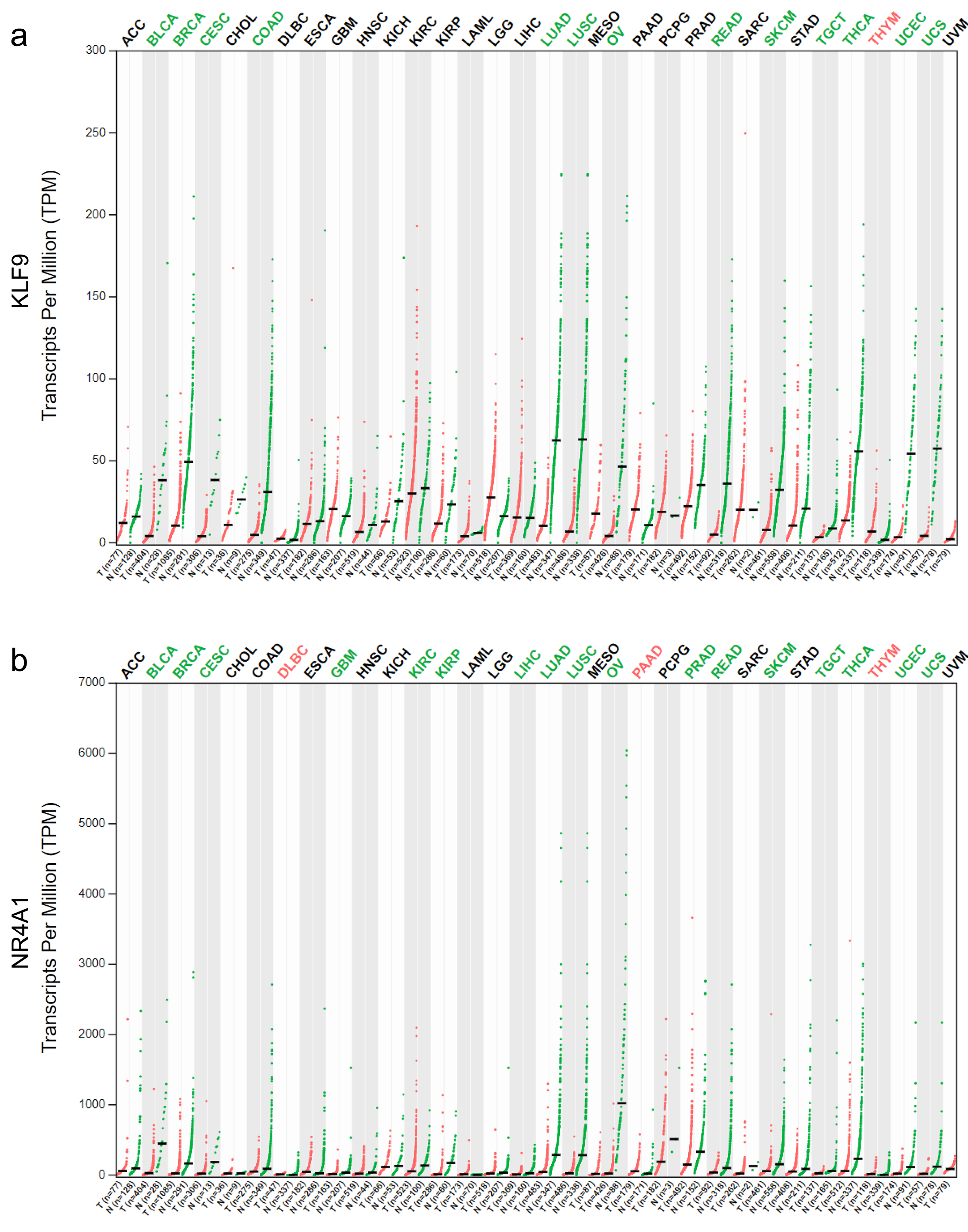


**a** The pan-cancer expression analysis for KLF9 using TCGA databases. The data was analyzed and visualized by GEPIA2 (http://gepia2.cancer-pku.cn/).

**b** The pan-cancer expression analysis for NR4A1 is similar in (**a**).

**Supplementary figure 7**


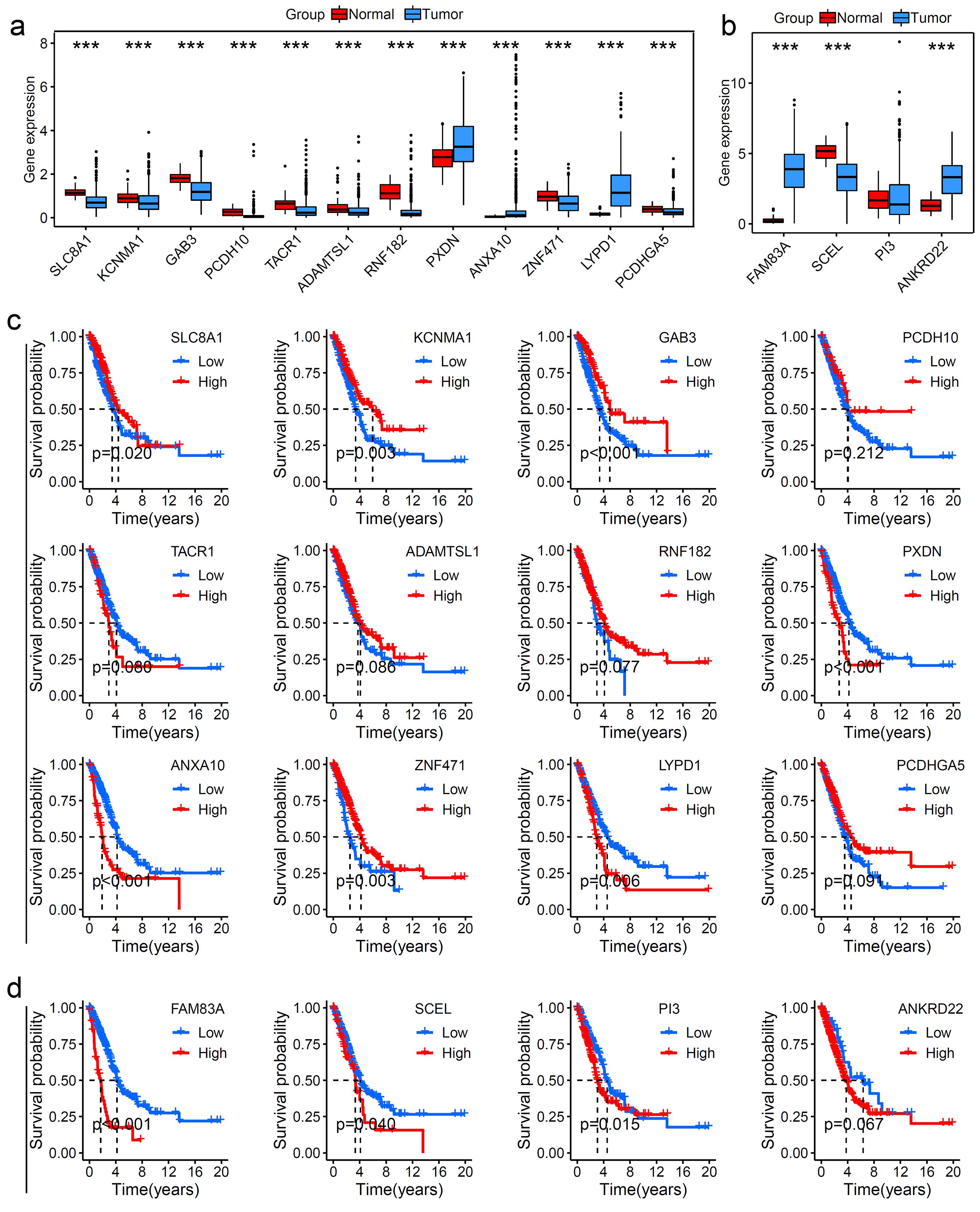


**a-b** The transcriptional level of corresponding genes in tumor and normal tissues using the TCGA LUAD dataset.

**c** The survival analysis for LUAD patients stratified by the expression level of genes in (**a**).

**d** The survival analysis for LUAD patients stratified by the expression level of genes in (**b**).

**P*< 0.05; ***P* < 0.01; ****P* < 0.001

| **Table S1. Gene primer used for qPCR.** | | |
| --- | --- | --- |
| Gene name | Forward primer (5'->3') | Reverse primer (5'->3') |
| ADAMTSL1 | GGCTTCCTGTGTCTAATGACC | TTAGGTGCTAGTTCAACAACCAG |
| RNF182 | GGTCAGTCCTTCTCACACG | GCAGTTCCACACAGTCCA |
| SLC8A1 | GACCTCGGTCCTAGCACCAT | ACACCAGGAGATATGACAGACAA |
| GALNT16 | ATCGCCATCCTGACCGTAG | CAAAGCCTTTCGAGGGAGTTC |
| GAB3 | GCCAAGAGATGACCCAAACAC | CCAGTCTCGCAGTTGGACAAA |
| PXDN | AGGATACCTAGTGGAGCATTTGA | CGAATCTGGGTCCAAAGTTTCTA |
| TENT5C | GGCCACGTTTTGGTCAAAGAC | GGGAACACAGAACCACATCTC |
| KCNMA1 | CTTGCAGGCTAATTCCCA | TGTGATGGATGGTTGACG |
| ANXA10 | TTGTGGAGACTATGTGCAAGGA | GGTATGCCTCTGCAATCATCAT |
| SLC16A2 | CCACGCCTACGGTAGAGAC | CAGAGTTATGGATGCCGAAGATG |
| PCDH10 | TGGATGGTGGAAGGAGTCTTT | TTCAGCGATATTCCCCACGAA |
| SCG2 | TCAACGATGAGATGAAACGCTC | TTTGCCCATTCTGTAACCTCC |
| ZNF471 | GCCTTCAGCCAAACTTCCAAT | AGCACAGGATGAGCTATCACTA |
| LYPD1 | GGCAACTTTTTGCGGATTGTT | CGTTCACCGTGCAATTCACA |
| DLC1 | CGCCTGAGCATCTACGA | TCTCCGACCACTGATTGAC |
| TACR1 | ATGCCCAGCAGAGTCGTGT | TCGTGGTAGCGGTCAGAGG |
| RASGRP3 | CTCTGCATGTATCGAAATGCCA | CTACTTCCCGAAATTCCTCAGTC |
| ZSCAN18 | GCGGGCTCATCCTCAATTCTT | CCTCTTCGGTCTTTGCTTCTC |
| ZNF365 | CCCATCTGGAGTTCAGTCACA | TTTTCCCGGTTTCAGGAGTTC |
| PCDHGA5 | TCGGAGACTTTCCAACTTGATTC | CTCCATCCTGAGCTACCACTT |
| CHL1 | ATGGAGCCGCTTTTACTTGGA | GGCAACTTGGACTTTTGACTGT |
| MAPK4 | CGGTGTCAATGGTTTGGTGC | GACGATGTTGTCGTGGTCCA |
| ONECUT3 | ATCCTGTGTCGCTCTCA | TGCTGCTCCTGTTCCTT |
| FAM83A | GGCCCTAAGGGACTGGACT | CACAGTGGCGCTGGATTTTT |
| SCEL | TCGGTACAGTTCTGATGACACT | AACATGGACATGCTCCTATTGG |
| PI3 | CACGGGAGTTCCTGTTAAAGG | TCTTTCAAGCAGCGGTTAGGG |
| FAM110B | TAGCTCCGAGGGCTCTAGC | CACCTTGCGGATGTCCGAA |
| TNFRSF19 | GACCTCAGCTCCACGAATATG | CACCCCACAACCAAGAGTCG |
| ANKRD22 | GCTCTTGTACGAATGCTACTTGA | CTCCGTGCAATATCCAGTGAG |
| C1orf116 | CAGTCTCAACCAGGTACACAC | TAGTCGGCTGTTTGGGTCCT |
| SMAD6 | GCTACCAACTCCCTCATCACT | CGTACACCGCATAGAGGCG |
| TGFB1 | GGCCAGATCCTGTCCAAGC | GTGGGTTTCCACCATTAGCAC |
| TGFBR3 | TGGGGTCTCCAGACTGTTTTT | CTGCTCCATACTCTTTTCGGG |
| MYC | TTCTCTGCTCTCCTCGACGGA GTC | CCTCTTTTCCACAGAAACAACATCGATTTCTTCC |
| WISP1 | AGTGGGTATGTGAGGACGA | GCTTGTGTAGGCTATGCAGTTC |
| ACTB | CTACCTCATGAAGATCCTCACCGA | TTCTCCTTAATGTCACGCACGATT |

| **Table S2. GSEA analysis in supplementary figure 4a** |  |  |  |
| --- | --- | --- | --- |
| Pathway | NES | NOM p-value | FDR q-value |
| ACTIVATION OF NF-kB-INDUCING KINASE ACTIVITY | -1.4340485 | 0 | 0.5884288 |
| CELLULAR RESPONSE TO IL6 | 1.3893563 | 0 | 0.75693345 |
| HETEROPHILIC_CELL_CELL_ADHESION_VIA_PLASMA_ MEMBRANE_CELL_ADHESION_MOLECULES | -1.4655042 | 0 | 0.5915728 |
| INNATE IMMUNE RESPONSE IN MUCOSA | 1.4437497 | 0 | 0.9622559 |
| NEGATIVE REGULATION OF CELL KILLING | -1.4625453 | 0 | 0.5730972 |
| NEGATIVE_REGULATION_OF_INTRINSIC_APOPTOTIC_ SIGNALING_PATHWAY_BY_P53_CLASS_MEDIATOR | -1.469997 | 0 | 0.6594822 |
| TYPE I INTERFERON RECEPTOR BINDING | 1.4287071 0.0952381 | | 0.93574136 |
| AUTOPHAGY - ANIMAL | 1.1852787 | 0 | 0.4203081 |
| DNA REPLICATION | -1.1815459 | 0 | 0.47889134 |
| FERROPTOSIS | 1.2248803 | 0.22198276 | 0.42046988 |
| NOTCH SIGNALING PATHWAY | -1.276643 | 0 | 0.5029995 |
| TGF-BETA SIGNALING PATHWAY | -1.1999633 | 0 | 0.4714519 |
|  |  |  |  |

| **Table S3. GSVA analysis in supplementary figure 4b** | | | | | | |
| --- | --- | --- | --- | --- | --- | --- |
| Signature | CFP1_sh_R1 | CFP1_sh_R2 | CFP1_sh_R3 | CFP1_NC_R1 | CFP1_NC_R2 | CFP1_NC_R3 |
| Angiogenesis | -0.299006 | -0.667683 | -0.803577 | 0.7745659 | -0.082906 | 0.6372396 |
| DNA replication | -0.469422 | -0.372473 | -0.374618 | 0.2630768 | 0.3295575 | 0.402034 |
| NER | -0.094264 | -0.317337 | -0.199282 | 0.0028241 | 0.2138651 | 0.279095 |
| Mismatch repair | -0.344241 | -0.129684 | -0.23551 | -0.021107 | 0.025374 | 0.3742098 |
| FGFR | -0.457715 | -0.785232 | -0.448146 | 0.5463245 | 0.6806291 | 0.367947 |
| WNT target | -9.93E-05 | -0.536763 | -0.242068 | -0.0509 | 0.2551394 | 0.490369 |
| EMT | -0.05798 | -0.310904 | -0.496398 | 0.3560968 | 0.085929 | 0.1774072 |
| Autophagy | 0.0686538 | 0.180441 | 0.1572446 | -0.287933 | -0.134213 | -0.333171 |
| Pyroptosis | 0.0392914 | 0.1104721 | 0.1938317 | 0.0590257 | -0.311682 | -0.017313 |
|  |  |  |  |  |  |  |

| **Table S4. Transcriptionally upregulated genes that had increased H3K4me3 deposition within their promoter regions simultaneously in the CFP1-knockdown cell lines.** | | | | | | |
| --- | --- | --- | --- | --- | --- | --- |
| id | ChIP_seq | | | RNA_seq | | |
|  | log2FC | logCPM | PValue | log2FC | pvalue | padj |
| TMCC1 | 1.336624 | 0.006945617 | 0.007594563 | 0.35667886 | 3.78E-06 | 4.37E-05 |
| ATP2C1 | 1.482521 | -0.239189552 | 0.007180229 | 0.39844989 | 7.11E-10 | 1.61E-08 |
| ZBTB38 | 1.062592 | 0.334943762 | 0.02079282 | 0.25244754 | 4.23E-07 | 5.97E-06 |
| PLSCR4 | 1.217497 | 1.061292493 | 0.001396556 | 0.37432345 | 0.002578857 | 0.01328372 |
| TM4SF18 | 1.048825 | 0.524573587 | 0.01486626 | 0.34624862 | 1.05E-05 | 0.000109847 |
| MBNL1 | 1.271116 | -0.141473315 | 0.01556371 | 0.35125202 | 2.19E-12 | 6.90E-11 |
| TIPARP | 1.597622 | 0.377433647 | 0.000560744 | 0.71278009 | 6.96E-14 | 2.62E-12 |
| TNIK | 1.580499 | 0.886778516 | 6.50E-05 | 1.06916842 | 2.75E-07 | 3.99E-06 |
| MACF1 | 1.18777 | 0.00740917 | 0.01669856 | 0.14642805 | 0.000812918 | 0.005050292 |
| P3H2 | 2.965978 | -0.66709318 | 7.68E-05 | 0.27102758 | 0.000253878 | 0.001855146 |
| GNPDA2 | 2.675728 | -0.537826699 | 0.000103608 | 0.28060329 | 0.01029941 | 0.04229257 |
| DCUN1D4 | 1.256624 | 0.311564604 | 0.006944527 | 0.21801678 | 0.007179687 | 0.03129072 |
| SLC2A1 | 1.210811 | -0.416494718 | 0.03709785 | 0.21472156 | 2.60E-05 | 0.000250374 |
| ABCG2 | 1.535059 | 0.333320599 | 0.00104138 | 0.47091962 | 8.68E-07 | 1.15E-05 |
| GPRIN3 | 2.171605 | -0.91049783 | 0.00423033 | 0.59300548 | 8.93E-34 | 1.30E-31 |
| TSPAN5 | 1.325705 | -0.342922401 | 0.01953014 | 0.60918051 | 0.007263483 | 0.03159693 |
| CISD2 | 1.299794 | -0.494601238 | 0.03063512 | 0.32549698 | 0.002630254 | 0.01349316 |
| ARSJ | 1.285663 | 1.153142584 | 0.000546742 | 1.45643614 | 2.90E-43 | 6.41E-41 |
| PCDH10 | 2.506646 | -0.66650781 | 0.000421961 | 1.88828108 | 0.000313195 | 0.002235603 |
| SLC7A11 | 1.49264 | 0.006482048 | 0.003182373 | 0.3778644 | 4.08E-18 | 2.25E-16 |
| SETD7 | 1.083712 | 2.275357433 | 0.000143472 | 0.23532519 | 2.27E-07 | 3.35E-06 |
| SCOC | 1.442424 | -0.141963738 | 0.006606612 | 0.61155137 | 6.36E-17 | 3.15E-15 |
| DCLK2 | 1.442424 | -0.141963738 | 0.006606612 | 0.46818049 | 3.29E-06 | 3.88E-05 |
| MAP9 | 1.315352 | 0.637351973 | 0.001708609 | 0.37631712 | 0.001360062 | 0.007799947 |
| ANXA10 | 1.418788 | 0.062094836 | 0.00408027 | 1.94970664 | 3.56E-06 | 4.15E-05 |
| PALLD | 1.147003 | 0.918813192 | 0.002699048 | 0.51957745 | 2.29E-26 | 2.28E-24 |
| SH3RF1 | 1.29188 | 0.167285227 | 0.00630869 | 0.40018674 | 8.49E-06 | 9.10E-05 |
| TRIML2 | 1.226619 | 0.759999594 | 0.00228741 | 1.01715421 | 4.21E-38 | 7.24E-36 |
| BASP1 | 1.005279 | 0.826415957 | 0.01010381 | 0.22267946 | 8.10E-07 | 1.08E-05 |
| RAI14 | 1.150042 | 0.24165084 | 0.01564512 | 0.35532099 | 1.20E-11 | 3.44E-10 |
| RICTOR | 1.464337 | 0.191782764 | 0.002779664 | 0.293872 | 1.31E-05 | 0.000133721 |
| EMB | 1.774005 | -0.174476339 | 0.001279284 | 0.94185564 | 2.80E-11 | 7.59E-10 |
| RAB3B | 1.032477 | 0.793916808 | 0.008956958 | 1.15768518 | 1.55E-52 | 5.07E-50 |
| IL6ST | 1.442684 | 0.35594853 | 0.001801679 | 0.4408561 | 7.36E-22 | 5.27E-20 |
| PDE4D | 1.32354 | 0.35635049 | 0.003978429 | 0.17112963 | 7.42E-05 | 0.000630358 |
| SCP2 | 1.121769 | 0.142355864 | 0.01753349 | 0.485136 | 8.49E-14 | 3.16E-12 |
| ZSWIM6 | 1.154583 | 0.463709744 | 0.008781508 | 0.28914933 | 0.005480493 | 0.02505478 |
| ERBIN | 1.286531 | 0.962570287 | 0.000715667 | 0.19095802 | 0.000874385 | 0.005376959 |
| OCLN | 1.007497 | 1.356361499 | 0.003774963 | 0.7874953 | 1.49E-07 | 2.26E-06 |
| MAP1B | 1.006999 | 0.777555151 | 0.01113754 | 0.33035656 | 1.01E-12 | 3.32E-11 |
| ARHGEF28 | 1.325705 | -0.342922401 | 0.01953014 | 0.26817727 | 4.25E-05 | 0.000386824 |
| F2RL1 | 1.062377 | 1.3224523 | 0.00251957 | 1.16826662 | 1.83E-140 | 4.27E-137 |
| LHFPL2 | 1.274559 | 0.543318677 | 0.003116237 | 0.24927198 | 0.000142901 | 0.001115559 |
| ARRDC3 | 1.456362 | -0.710318659 | 0.0276789 | 0.28160098 | 0.000817228 | 0.005075139 |
| CAST | 1.210811 | -0.416494718 | 0.03709785 | 0.1907153 | 5.89E-06 | 6.48E-05 |
| CSNK1G3 | 3.464178 | -0.760391441 | 2.95E-05 | 0.20794042 | 0.006540715 | 0.02891051 |
| FBN2 | 1.077881 | 0.87335759 | 0.005269081 | 0.3735508 | 5.59E-12 | 1.68E-10 |
| RAPGEF6 | 1.086673 | 0.777222708 | 0.006295613 | 0.66107618 | 0.00025931 | 0.001890615 |
| ETF1 | 1.030805 | 0.242072767 | 0.02339332 | 0.12951302 | 0.006883972 | 0.03015475 |
| PCDHGA5 | 1.043013 | 1.345063139 | 0.002824693 | 1.5481554 | 0.00788578 | 0.03371657 |
| ROR1 | 1.044852 | 0.007872704 | 0.03399614 | 0.500098 | 8.54E-05 | 0.000712095 |
| SYNPO | 1.162556 | 0.977567582 | 0.002886596 | 0.42613316 | 7.02E-19 | 4.06E-17 |
| PDE4B | 1.035259 | 0.743923839 | 0.009865202 | 0.18693691 | 0.001316869 | 0.007597572 |
| SH3PXD2B | 1.185525 | 0.484029715 | 0.006804463 | 0.18912112 | 0.005693294 | 0.02587559 |
| TMEM170B | 1.49264 | 0.006482048 | 0.003182373 | 0.38742492 | 0.006313324 | 0.02812252 |
| RNF182 | 1.989591 | -0.344503658 | 0.000857787 | 3.18251295 | 1.91E-23 | 1.49E-21 |
| GNG12 | 1.089074 | 0.035702225 | 0.02594261 | 0.54052384 | 3.06E-31 | 4.03E-29 |
| MIGA1 | 1.210811 | -0.416494718 | 0.03709785 | 0.28752721 | 0.000203143 | 0.00152605 |
| DST | 1.537261 | 0.791937213 | 0.000150034 | 0.86964297 | 4.60E-76 | 2.89E-73 |
| PRKACB | 1.591325 | 0.542207632 | 0.000289093 | 0.37085361 | 0.000508766 | 0.003395719 |
| KCNQ5 | 1.870979 | 0.033409458 | 0.000296643 | 1.33436297 | 1.44E-06 | 1.82E-05 |
| CD109 | 2.132292 | -0.665922413 | 0.001822828 | 0.14122473 | 0.005808212 | 0.02631006 |
| NT5E | 1.32354 | 0.35635049 | 0.003978429 | 0.81611073 | 1.23E-39 | 2.23E-37 |
| MANEA | 1.272343 | 0.062548461 | 0.009315432 | 0.50558971 | 1.87E-05 | 0.000184857 |
| DDAH1 | 1.94474 | 0.087547848 | 0.000142624 | 0.43966812 | 6.01E-05 | 0.000523508 |
| AMD1 | 1.586948 | -0.455841535 | 0.008412736 | 0.19550416 | 9.61E-05 | 0.000788665 |
| SH3GLB1 | 2.251953 | -0.578882011 | 0.000758987 | 0.32461239 | 5.95E-06 | 6.53E-05 |
| EPB41L2 | 1.395315 | -0.173483967 | 0.009165782 | 0.22178791 | 0.004996944 | 0.02313547 |
| PDE7B | 1.010842 | 0.674566487 | 0.01362799 | 0.89237429 | 2.30E-17 | 1.17E-15 |
| MAP7 | 1.776471 | -0.495710713 | 0.004181329 | 0.37060458 | 2.86E-11 | 7.72E-10 |
| LRRC8D | 1.325705 | -0.342922401 | 0.01953014 | 0.24712108 | 0.000810162 | 0.005037002 |
| UTRN | 1.228118 | 0.44271074 | 0.00575891 | 0.37185185 | 2.49E-09 | 5.25E-08 |
| ZNF12 | 1.121769 | 0.142355864 | 0.01753349 | 0.27562921 | 0.004624559 | 0.02166304 |
| TSPAN13 | 2.349261 | -0.27499762 | 9.83E-05 | 0.26090557 | 0.000940605 | 0.005698386 |
| PDE1C | 1.870979 | 0.033409458 | 0.000296643 | 1.03288256 | 6.57E-33 | 9.26E-31 |
| GCLM | 1.643251 | -0.417575837 | 0.005823769 | 0.37261893 | 5.68E-14 | 2.15E-12 |
| BMPER | 1.259877 | 1.775311632 | 8.25E-05 | 0.56877497 | 5.74E-11 | 1.49E-09 |
| STARD3NL | 1.156766 | -0.110151549 | 0.02513009 | 0.27622813 | 0.002427268 | 0.01267812 |
| ABCD3 | 1.643251 | -0.417575837 | 0.005823769 | 0.49257938 | 6.40E-09 | 1.25E-07 |
| TNS3 | 1.057665 | -0.172491634 | 0.04444927 | 0.12195456 | 0.004031334 | 0.01928784 |
| GRB10 | 1.243986 | -0.272426238 | 0.02384497 | 0.55383865 | 1.14E-23 | 9.29E-22 |
| SEC61G | 1.029888 | 0.842234296 | 0.008150169 | 0.21817475 | 0.009004084 | 0.03773217 |
| EGFR | 3.426868 | 2.528711903 | 4.58E-25 | 0.13599661 | 0.001647306 | 0.009182986 |
| HIP1 | 1.456362 | -0.710318659 | 0.0276789 | 0.16131874 | 0.002570247 | 0.0132519 |
| MAGI2-AS3 | 1.305605 | 0.421404229 | 0.003663412 | 1.09748018 | 6.90E-07 | 9.32E-06 |
| SEMA3C | 1.03996 | 1.075599343 | 0.005883008 | 0.16714817 | 0.000848102 | 0.00524693 |
| SAMD9 | 5.34873 | 0.029281325 | 3.89E-11 | 0.54848682 | 4.14E-09 | 8.33E-08 |
| SMURF1 | 1.261034 | 0.600616732 | 0.002879112 | 0.20955071 | 0.003181581 | 0.01586324 |
| DLD | 1.45818 | 0.088894435 | 0.002976471 | 0.27813333 | 6.71E-07 | 9.09E-06 |
| NRCAM | 1.131098 | 0.379026348 | 0.01256134 | 0.84237227 | 2.96E-77 | 2.02E-74 |
| MET | 4.353151 | 4.648429458 | 1.90E-56 | 0.14746986 | 0.002890584 | 0.01462206 |
| CAPZA2 | 2.928692 | 3.099411539 | 1.95E-24 | 0.26125117 | 2.41E-06 | 2.92E-05 |
| FAM3C | 1.285308 | 1.239745561 | 0.000397981 | 0.60999448 | 2.10E-11 | 5.80E-10 |
| CREB3L2 | 1.442424 | -0.141963738 | 0.006606612 | 0.62763184 | 2.00E-28 | 2.32E-26 |
| HIPK2 | 2.675728 | -0.537826699 | 0.000103608 | 0.56784926 | 1.12E-41 | 2.24E-39 |
| SLC16A4 | 1.475759 | -0.379770586 | 0.01104842 | 0.52157197 | 1.41E-23 | 1.14E-21 |
| DLC1 | 1.189598 | 2.365640167 | 2.39E-05 | 1.75641918 | 3.38E-33 | 4.80E-31 |
| CSGALNACT1 | 2.012278 | 0.854359821 | 1.13E-06 | 0.2379444 | 1.85E-08 | 3.31E-07 |
| NRG1 | 1.035407 | 1.154247126 | 0.004986264 | 0.1688966 | 0.000474326 | 0.003202485 |
| TENT5C | 1.533876 | 0.141037382 | 0.001570614 | 2.08854157 | 0.000248237 | 0.001816365 |
| GEM | 1.632575 | -0.273454779 | 0.003819578 | 1.04954681 | 3.11E-09 | 6.38E-08 |
| EFR3A | 1.069441 | 0.656428549 | 0.009571636 | 0.1970408 | 0.003422103 | 0.01683642 |
| KANK1 | 1.308778 | 0.26491814 | 0.005758989 | 0.81788251 | 2.52E-26 | 2.49E-24 |
| SMARCA2 | 1.230676 | 0.035243708 | 0.01249671 | 0.2243252 | 0.004603777 | 0.02159044 |
| ADAMTSL1 | 1.02708 | 0.312383973 | 0.02660856 | 4.48241822 | 2.88E-07 | 4.16E-06 |
| UBAP1 | 1.005279 | 0.826415957 | 0.01010381 | 0.29364275 | 0.000261241 | 0.001900451 |
| LINC01474 | 1.143549 | -0.020976162 | 0.02221906 | 0.63888527 | 0.003360989 | 0.01659571 |
| GOLM1 | 1.532266 | -0.080927638 | 0.003394765 | 0.41717897 | 1.29E-17 | 6.74E-16 |
| ZNF510 | 1.43212 | -0.272940507 | 0.01006829 | 0.40602584 | 0.005160305 | 0.02380413 |
| ABCA1 | 1.131098 | 0.379026348 | 0.01256134 | 0.60138266 | 1.05E-20 | 6.81E-19 |
| EPB41L4B | 1.769402 | 0.823491829 | 1.49E-05 | 0.39070805 | 1.38E-05 | 0.000139963 |
| SUSD1 | 1.115556 | 0.504442896 | 0.01019156 | 0.48761258 | 6.25E-05 | 0.000539506 |
| PAPPA | 1.261034 | 0.600616732 | 0.002879112 | 0.41421324 | 2.42E-27 | 2.55E-25 |
| SNX27 | 1.186451 | 0.265335934 | 0.01197492 | 0.21270499 | 0.002603823 | 0.01338699 |
| STOM | 1.052993 | 0.464093424 | 0.01657789 | 0.60175069 | 6.16E-34 | 9.06E-32 |
| FAM102A | 1.203876 | -0.079969011 | 0.01875381 | 0.25466993 | 0.000237891 | 0.001750071 |
| FNBP1 | 1.711079 | -0.081406973 | 0.001255385 | 0.47544333 | 1.65E-07 | 2.50E-06 |
| PITRM1 | 1.048362 | 1.742830614 | 0.001054123 | 0.22085749 | 0.00023201 | 0.001716856 |
| LINC00707 | 1.068157 | 0.265753703 | 0.02339332 | 0.47885642 | 3.02E-05 | 0.000284758 |
| SFMBT2 | 1.721604 | 0.989716715 | 9.38E-06 | 0.82840047 | 6.01E-27 | 6.21E-25 |
| FAM171A1 | 1.531221 | -0.206214404 | 0.005100126 | 0.41425182 | 3.82E-06 | 4.40E-05 |
| JCAD | 1.204913 | 1.005921283 | 0.001862905 | 1.48922613 | 2.82E-92 | 3.29E-89 |
| NRP1 | 1.070213 | 0.963481894 | 0.006216513 | 0.44660361 | 4.47E-32 | 6.03E-30 |
| LINC00839 | 1.073477 | 2.754671418 | 5.63E-05 | 3.31585974 | 1.90E-05 | 0.00018701 |
| ARID5B | 1.711079 | -0.081406973 | 0.001255385 | 0.94894506 | 5.79E-09 | 1.14E-07 |
| RTKN2 | 1.35587 | 0.378230051 | 0.002998891 | 1.41930004 | 0.00031316 | 0.002235603 |
| ZNF365 | 2.965978 | -0.66709318 | 7.68E-05 | 1.63043165 | 0.009791859 | 0.04053438 |
| DNAJC12 | 1.030805 | 0.242072767 | 0.02339332 | 0.7843864 | 5.12E-14 | 1.96E-12 |
| CHST3 | 1.074346 | 0.193074397 | 0.02036499 | 0.3924397 | 1.54E-09 | 3.32E-08 |
| KCNMA1 | 1.186451 | 0.265335934 | 0.01197492 | 1.9558261 | 1.01E-43 | 2.33E-41 |
| PPP1R3C | 1.119787 | -0.238173371 | 0.03830181 | 0.57095922 | 7.69E-11 | 1.96E-09 |
| PLCE1 | 1.003656 | 1.888139232 | 0.001148951 | 0.90731031 | 8.09E-16 | 3.68E-14 |
| BLOC1S2 | 1.131098 | 0.379026348 | 0.01256134 | 0.4463372 | 9.35E-08 | 1.48E-06 |
| AFAP1L2 | 2.513773 | -0.381905508 | 8.22E-05 | 0.98510178 | 1.24E-19 | 7.66E-18 |
| ATE1 | 1.272343 | 0.062548461 | 0.009315432 | 0.29374112 | 4.31E-05 | 0.000391082 |
| ADAM12 | 1.368343 | 0.856600491 | 0.00051059 | 0.72655963 | 5.39E-09 | 1.07E-07 |
| DOCK1 | 1.60108 | -0.806651179 | 0.02114381 | 0.16648737 | 0.008278909 | 0.03514396 |
| AMPD3 | 1.204913 | 1.005921283 | 0.001862905 | 1.05527318 | 1.47E-13 | 5.31E-12 |
| MICAL2 | 1.039605 | 0.09024084 | 0.02990678 | 0.26291803 | 1.85E-06 | 2.30E-05 |
| TEAD1 | 1.401153 | 0.240806914 | 0.003497867 | 0.15892497 | 3.63E-05 | 0.000335977 |
| C11orf58 | 1.271116 | -0.141473315 | 0.01556371 | 0.28882641 | 1.16E-07 | 1.80E-06 |
| NAV2 | 1.452022 | 1.398024063 | 3.51E-05 | 1.21834997 | 5.55E-14 | 2.11E-12 |
| BDNF | 1.100394 | 1.115177727 | 0.00322592 | 0.79508271 | 1.26E-23 | 1.02E-21 |
| CAT | 1.225633 | 1.019890992 | 0.001493118 | 0.26605162 | 2.59E-05 | 0.000249947 |
| ALX4 | 1.496524 | 0.115202709 | 0.00216503 | 0.33593307 | 0.000261034 | 0.001899903 |
| PTPRJ | 1.070728 | 1.216483183 | 0.003132815 | 0.15673052 | 0.000671099 | 0.004298348 |
| RTN4RL2 | 1.502729 | 0.310745133 | 0.001414816 | 0.46660976 | 8.98E-13 | 2.99E-11 |
| CEP126 | 1.271116 | -0.141473315 | 0.01556371 | 0.53137048 | 2.95E-05 | 0.000279821 |
| ACAT1 | 1.332779 | 1.140136305 | 0.000367518 | 0.30266894 | 0.000567825 | 0.0037215 |
| DDX10 | 1.378291 | 0.034785174 | 0.005576031 | 0.48779332 | 3.89E-10 | 9.15E-09 |
| MIR100HG | 1.448726 | 0.44193624 | 0.001266002 | 0.4118311 | 3.64E-05 | 0.000336933 |
| UBASH3B | 1.041495 | 0.638416278 | 0.01205052 | 0.16578938 | 0.000531369 | 0.003524983 |
| KIRREL3 | 1.418212 | 0.708117879 | 0.000588384 | 0.99844097 | 2.54E-19 | 1.54E-17 |
| PLEKHG6 | 1.097929 | -0.04993457 | 0.02943072 | 1.16244648 | 3.07E-22 | 2.27E-20 |
| GRIN2B | 1.939189 | -0.380838054 | 0.00128011 | 1.05390912 | 7.68E-09 | 1.48E-07 |
| BCAT1 | 1.042443 | 1.034606173 | 0.006406987 | 0.86091088 | 1.78E-23 | 1.41E-21 |
| ARNTL2 | 1.253432 | 0.888039533 | 0.001226077 | 0.32452707 | 6.33E-06 | 6.93E-05 |
| SRGAP1 | 1.054067 | 1.849021135 | 0.000729458 | 0.31540358 | 5.15E-05 | 0.000455958 |
| PHLDA1 | 1.045006 | 0.582618328 | 0.0133673 | 0.26451498 | 2.06E-08 | 3.65E-07 |
| NTN4 | 1.174738 | 0.33453801 | 0.01077289 | 0.14328316 | 0.009640652 | 0.04001997 |
| NUAK1 | 1.191311 | 0.948336848 | 0.001712831 | 0.20766798 | 0.009979892 | 0.04114597 |
| FBXO21 | 1.104566 | 0.289050109 | 0.01813015 | 0.17710855 | 0.000827875 | 0.005127615 |
| WSB2 | 1.56668 | 0.355546544 | 0.000764908 | 0.19638364 | 0.001175719 | 0.006908944 |
| STARD13 | 1.776471 | -0.495710713 | 0.004181329 | 0.89958111 | 6.00E-07 | 8.24E-06 |
| PCDH9 | 1.403516 | -0.577173012 | 0.02463569 | 0.36077711 | 1.73E-11 | 4.82E-10 |
| SLITRK6 | 2.081829 | 0.190060263 | 4.71E-05 | 0.5315587 | 0.001108582 | 0.006565104 |
| GPC6 | 1.586948 | -0.455841535 | 0.008412736 | 0.43501827 | 0.00055428 | 0.003647357 |
| MBNL2 | 1.081272 | 0.116535005 | 0.02294735 | 0.40547446 | 3.09E-07 | 4.44E-06 |
| ATP2B4 | 1.427928 | 0.166850243 | 0.002779664 | 0.30018591 | 7.64E-05 | 0.000646541 |
| ABHD4 | 1.02708 | 0.312383973 | 0.02660856 | 0.61484894 | 2.01E-17 | 1.03E-15 |
| G2E3 | 1.684634 | 0.140597845 | 0.000602715 | 0.32138752 | 0.000336767 | 0.002382557 |
| MAP4K5 | 1.312841 | 0.089343257 | 0.006918611 | 0.23523591 | 0.007623298 | 0.03283001 |
| FERMT2 | 1.378291 | 0.034785174 | 0.005576031 | 0.18392288 | 0.01072384 | 0.0436623 |
| PELI2 | 1.115556 | 0.504442896 | 0.01019156 | 0.39667486 | 0.004435615 | 0.02094211 |
| GALNT16 | 1.657585 | 0.420231661 | 0.000299766 | 2.36642216 | 1.20E-24 | 1.06E-22 |
| PNMA1 | 1.982689 | -0.023319734 | 0.000196752 | 0.31633267 | 1.90E-07 | 2.84E-06 |
| GPATCH2L | 1.390577 | 0.141476898 | 0.003778445 | 0.38908635 | 4.30E-08 | 7.25E-07 |
| DIO2 | 1.657729 | 0.741558944 | 6.49E-05 | 0.81032695 | 5.95E-17 | 2.95E-15 |
| C14orf132 | 1.035407 | 1.154247126 | 0.004986264 | 0.60487258 | 1.31E-05 | 0.000134207 |
| EML1 | 1.442424 | -0.141963738 | 0.006606612 | 0.2056479 | 0.01223644 | 0.04857883 |
| GABRA5 | 1.012998 | 0.620175105 | 0.01513057 | 0.29495579 | 4.40E-08 | 7.40E-07 |
| C15orf41 | 2.907388 | -0.712693194 | 0.000126105 | 0.47236328 | 0.002387429 | 0.01250263 |
| FBN1 | 1.249496 | -0.050408498 | 0.01393199 | 0.60971081 | 6.15E-24 | 5.15E-22 |
| ATP8B4 | 3.170614 | 1.209790954 | 7.81E-14 | 1.44828159 | 1.98E-11 | 5.49E-10 |
| MYO5C | 2.03242 | 0.869863438 | 8.27E-07 | 0.21873983 | 0.00381506 | 0.01839312 |
| ANXA2 | 1.331003 | 0.581526577 | 0.001829976 | 0.26563996 | 1.85E-20 | 1.19E-18 |
| USP3 | 1.015336 | 0.563650915 | 0.01684261 | 0.27462756 | 0.000410124 | 0.002829822 |
| NOX5 | 1.134037 | 1.998124914 | 0.000177751 | 1.44996417 | 0.001968579 | 0.01065759 |
| UACA | 1.486762 | 1.541054647 | 1.17E-05 | 0.46097141 | 6.47E-08 | 1.05E-06 |
| ETFA | 1.271116 | -0.141473315 | 0.01556371 | 0.12562717 | 0.009537322 | 0.03966157 |
| TBC1D2B | 1.409146 | 1.791082794 | 1.15E-05 | 0.23026696 | 0.005702154 | 0.02590865 |
| MFGE8 | 1.947744 | 0.440386914 | 2.89E-05 | 1.11565403 | 3.28E-100 | 4.46E-97 |
| CACNA1H | 1.243986 | -0.272426238 | 0.02384497 | 0.16276452 | 0.00704048 | 0.03077432 |
| SNX29 | 1.744559 | -0.71091234 | 0.009625718 | 0.33423194 | 0.004647166 | 0.02173777 |
| NOMO1 | 1.697439 | -0.380304322 | 0.004015236 | 0.19849392 | 0.002511261 | 0.01302173 |
| CAPN2 | 2.225877 | 0.004627595 | 3.79E-05 | 0.22634081 | 3.90E-08 | 6.62E-07 |
| FTO | 1.004174 | 1.452859388 | 0.003043045 | 0.22368579 | 0.003151065 | 0.0157351 |
| MMP2 | 1.328275 | 0.991215187 | 0.000442871 | 0.9990613 | 1.37E-23 | 1.11E-21 |
| CES1 | 1.047889 | 0.948948932 | 0.007641582 | 1.19227708 | 2.58E-136 | 5.27E-133 |
| ZFHX3 | 1.303057 | 0.562549726 | 0.002390599 | 0.28282824 | 3.14E-07 | 4.50E-06 |
| HSBP1 | 2.040814 | 0.286153727 | 3.55E-05 | 0.21107963 | 0.000445433 | 0.003041344 |
| GALNT2 | 1.532266 | -0.080927638 | 0.003394765 | 0.38064948 | 1.38E-21 | 9.57E-20 |
| PMP22 | 1.017725 | 3.604042984 | 2.35E-05 | 0.30117088 | 2.73E-07 | 3.97E-06 |
| TOM1L2 | 1.084614 | 0.484409958 | 0.01301803 | 0.21808345 | 0.001853076 | 0.01013979 |
| WSB1 | 1.818227 | -0.142944617 | 0.000883481 | 0.28637633 | 1.00E-05 | 0.000105789 |
| SLFN5 | 1.726611 | 0.377035405 | 0.000219377 | 0.57448703 | 2.82E-16 | 1.34E-14 |
| TMEM92 | 1.176927 | 1.034019527 | 0.002156652 | 0.52074128 | 8.52E-06 | 9.13E-05 |
| SPAG9 | 1.200709 | 2.133237341 | 4.65E-05 | 0.14074109 | 0.000899834 | 0.005518912 |
| EDARADD | 1.121769 | 0.142355864 | 0.01753349 | 0.67451813 | 0.006645588 | 0.02931636 |
| GNA13 | 1.101291 | 0.888669877 | 0.004223812 | 0.17150103 | 0.00425686 | 0.02024238 |
| LINC01139 | 1.380038 | 2.145214836 | 3.35E-06 | 1.2658016 | 0.002143612 | 0.01145714 |
| TIMP2 | 1.273266 | 0.241228889 | 0.007645969 | 0.1546117 | 0.000644098 | 0.004151444 |
| LPIN2 | 1.230676 | 0.035243708 | 0.01249671 | 0.26866537 | 0.006537517 | 0.02891041 |
| GNAL | 1.057665 | -0.172491634 | 0.04444927 | 0.33344078 | 0.002041418 | 0.01100426 |
| ANKRD29 | 1.488044 | -0.111121152 | 0.004744033 | 0.76728786 | 1.46E-10 | 3.64E-09 |
| LAMA3 | 1.363845 | -0.080448318 | 0.008342081 | 0.31250778 | 1.82E-06 | 2.26E-05 |
| CDH2 | 1.081272 | 0.116535005 | 0.02294735 | 0.80644575 | 2.47E-15 | 1.07E-13 |
| TMX3 | 1.774005 | -0.174476339 | 0.001279284 | 0.66527466 | 3.40E-22 | 2.50E-20 |
| PXDN | 1.900278 | 0.598451191 | 1.68E-05 | 2.18055644 | 2.70E-89 | 2.59E-86 |
| DIRAS1 | 1.041495 | 0.638416278 | 0.01205052 | 0.53267682 | 0.004560485 | 0.02143664 |
| CD70 | 1.295366 | 1.114332756 | 0.000576244 | 0.8317727 | 0.001440806 | 0.008168403 |
| MBOAT2 | 1.293718 | -0.021444844 | 0.01030639 | 0.4722947 | 1.80E-06 | 2.24E-05 |
| ITGB1BP1 | 1.236857 | 0.217142003 | 0.01011843 | 0.1824834 | 0.01188565 | 0.04739647 |
| ZNF738 | 1.027218 | 2.477679265 | 0.000197249 | 1.18925627 | 0.01181849 | 0.04719984 |
| RHPN2 | 1.383711 | 0.808784653 | 0.000535418 | 0.26815993 | 5.85E-07 | 8.05E-06 |
| ZFP14 | 2.105203 | -0.456936623 | 0.000819566 | 0.40536204 | 0.005829848 | 0.02638612 |
| ZNF420 | 1.156766 | -0.110151549 | 0.02513009 | 1.1650275 | 1.16E-06 | 1.49E-05 |
| ZNF527 | 1.199505 | 0.192643876 | 0.01011843 | 0.51978058 | 0.007956677 | 0.03396138 |
| NOVA2 | 2.028625 | -0.143435073 | 0.000273436 | 1.10863512 | 0.00268349 | 0.01371459 |
| GREB1 | 1.49264 | 0.006482048 | 0.003182373 | 0.4197465 | 0.01188755 | 0.04739647 |
| ZNF480 | 1.456362 | -0.710318659 | 0.0276789 | 0.43811945 | 0.000169055 | 0.001294976 |
| ZNF471 | 1.188077 | 2.617457867 | 1.47E-05 | 1.8340358 | 0.000850192 | 0.005257868 |
| ZSCAN18 | 1.071096 | 1.431648168 | 0.001711227 | 1.63975687 | 0.001857341 | 0.01015972 |
| KIF16B | 1.131098 | 0.379026348 | 0.01256134 | 0.30424048 | 0.000156815 | 0.001212196 |
| PHF20 | 1.728385 | -0.206716478 | 0.001847602 | 0.22131856 | 0.001060256 | 0.006313238 |
| TOP1 | 1.253432 | 0.888039533 | 0.001226077 | 0.19895946 | 0.002146998 | 0.01147149 |
| PREX1 | 1.274559 | 0.543318677 | 0.003116237 | 0.56226611 | 1.06E-07 | 1.66E-06 |
| PMEPA1 | 1.336795 | -0.620010046 | 0.03484493 | 0.17422135 | 8.55E-05 | 0.000712654 |
| RUNX1 | 1.403516 | -0.577173012 | 0.02463569 | 0.33778239 | 0.001378839 | 0.007891015 |
| ETS2 | 1.936121 | -0.578312363 | 0.00285251 | 0.40130249 | 8.05E-07 | 1.07E-05 |
| CLIP4 | 1.006867 | 1.793040722 | 0.001430792 | 0.16563605 | 0.01043396 | 0.04271625 |
| SHISAL1 | 1.65681 | -0.577742697 | 0.009038249 | 0.58960169 | 0.005888694 | 0.02660823 |
| SH3KBP1 | 1.050819 | -0.079489718 | 0.03879354 | 0.33977728 | 2.23E-08 | 3.94E-07 |
| RPS6KA3 | 1.511203 | 0.636642295 | 0.000366334 | 0.12742443 | 0.004036758 | 0.01930665 |
| LTBP1 | 1.498129 | 2.806054715 | 3.16E-08 | 0.60125574 | 8.97E-18 | 4.82E-16 |
| RASGRP3 | 1.769402 | 0.823491829 | 1.49E-05 | 1.6584888 | 0.000859393 | 0.005302727 |
| MSN | 1.726611 | 0.377035405 | 0.000219377 | 0.1226756 | 0.001048038 | 0.006245503 |
| EDA2R | 1.365641 | 0.216715787 | 0.004704587 | 0.38875221 | 0.000151779 | 0.001177054 |
| AR | 4.340002 | 0.497655057 | 7.03E-13 | 0.54598407 | 1.59E-14 | 6.41E-13 |
| SLC16A2 | 1.272343 | 0.062548461 | 0.009315432 | 1.93695947 | 7.82E-82 | 6.08E-79 |
| RPS6KA6 | 2.557945 | -0.113545377 | 1.15E-05 | 0.39714588 | 0.007777794 | 0.03336345 |
| APOOL | 4.583983 | 0.719142409 | 3.86E-15 | 0.31682352 | 0.000585472 | 0.003821823 |
| KLHL4 | 1.578331 | -0.173980148 | 0.003609386 | 0.92303839 | 1.70E-06 | 2.13E-05 |
| SRPX2 | 1.623951 | -0.142454172 | 0.002545854 | 0.87718932 | 2.59E-06 | 3.12E-05 |
| FEZ2 | 1.253536 | 0.141916392 | 0.008432619 | 0.25997797 | 4.38E-05 | 0.00039616 |
| DOCK11 | 1.236023 | -0.535016237 | 0.04253884 | 0.3675806 | 0.01238337 | 0.04907396 |
| STAG2 | 1.467286 | -0.53557836 | 0.01731167 | 0.17235327 | 0.001540513 | 0.008637765 |
| STRN | 1.236857 | 0.217142003 | 0.01011843 | 0.31291908 | 2.90E-06 | 3.46E-05 |
| EIF2AK2 | 1.038256 | 0.692134963 | 0.01089032 | 0.36619892 | 2.27E-14 | 8.98E-13 |
| CDC42EP3 | 1.222416 | -0.172987796 | 0.0211185 | 1.03509141 | 4.33E-37 | 7.29E-35 |
| GAB3 | 1.572529 | 0.06164119 | 0.001648005 | 2.30453769 | 0.000297263 | 0.002136812 |
| VBP1 | 1.143549 | -0.020976162 | 0.02221906 | 0.25147884 | 0.000963616 | 0.005816224 |
| SLC8A1 | 1.496524 | 0.115202709 | 0.00216503 | 2.49200015 | 0.000547372 | 0.003609163 |
| MCFD2 | 1.18389 | 0.991814388 | 0.002320767 | 0.29142245 | 1.12E-13 | 4.10E-12 |
| FOXN2 | 2.740222 | 0.031574915 | 1.33E-06 | 0.22551902 | 0.006109264 | 0.02740781 |
| RTN4 | 1.49985 | 0.216289547 | 0.002039309 | 0.23406031 | 9.35E-11 | 2.37E-09 |
| VRK2 | 1.251874 | 1.298713642 | 0.000446108 | 0.40247831 | 7.11E-06 | 7.75E-05 |
| UGP2 | 1.759509 | 1.017822455 | 5.37E-06 | 0.15818665 | 0.003355643 | 0.01657681 |
| TACR1 | 1.161161 | 0.167720189 | 0.0133442 | 1.6803003 | 4.02E-14 | 1.55E-12 |
| INPP4A | 1.336624 | 0.006945617 | 0.007594563 | 0.21354083 | 0.003113745 | 0.01558208 |
| MAP4K4 | 1.523609 | -0.857028529 | 0.0310526 | 0.23783561 | 1.02E-05 | 0.000107281 |
| ST6GAL2 | 1.023716 | 0.379424457 | 0.0236259 | 0.24382313 | 0.001445517 | 0.008189423 |
| LIMS1 | 1.870979 | 0.033409458 | 0.000296643 | 0.19863795 | 0.001107629 | 0.00656184 |
| LUZP1 | 1.266839 | -0.664166057 | 0.04895303 | 0.25561725 | 0.000163222 | 0.001253828 |
| DDX18 | 6.780378 | 1.368362291 | 8.15E-26 | 0.13483994 | 0.008797501 | 0.03696127 |
| MAP3K2 | 1.800068 | -0.308545917 | 0.001888542 | 0.2393766 | 0.000486421 | 0.003270628 |
| LYPD1 | 1.048825 | 0.524573587 | 0.01486626 | 1.81623525 | 0.000844485 | 0.005228509 |
| NCKAP5 | 1.249496 | -0.050408498 | 0.01393199 | 0.69812943 | 6.18E-06 | 6.78E-05 |
| KYNU | 1.081272 | 0.116535005 | 0.02294735 | 0.1729014 | 1.11E-05 | 0.00011632 |
| GTDC1 | 1.479352 | 0.990615878 | 0.000106442 | 0.52702848 | 0.000237422 | 0.001748297 |
| TEX41 | 1.091641 | 1.969654534 | 0.000328173 | 1.23589033 | 1.65E-08 | 2.98E-07 |
| RND3 | 1.318226 | -0.110636344 | 0.01141828 | 0.87769525 | 2.83E-29 | 3.47E-27 |
| CACNB4 | 4.23362 | -0.115484978 | 4.67E-09 | 0.7526778 | 0.01138404 | 0.04576864 |
| GALNT13 | 1.3629 | 1.114051048 | 0.00030576 | 0.61001222 | 9.27E-13 | 3.07E-11 |
| GPD2 | 1.082121 | 0.826091166 | 0.005753904 | 0.17398847 | 0.002534032 | 0.01311069 |
| TANK | 1.190246 | 1.088342821 | 0.001621181 | 0.33943519 | 9.72E-05 | 0.000796378 |
| FIGN | 1.818227 | -0.142944617 | 0.000883481 | 0.63076516 | 0.000141916 | 0.001110012 |
| SCN9A | 1.529947 | -0.343449488 | 0.007786219 | 0.4978968 | 0.000533076 | 0.003533441 |
| GORASP2 | 1.505247 | 0.39938662 | 0.000993175 | 0.21333501 | 0.000200762 | 0.00150955 |
| WIPF1 | 1.65681 | -0.577742697 | 0.009038249 | 0.28679116 | 0.007359705 | 0.0318797 |
| TFPI | 1.214144 | 0.116090927 | 0.01123289 | 0.377662 | 5.47E-13 | 1.87E-11 |
| SLC40A1 | 1.057513 | 0.810094106 | 0.007186815 | 1.08875319 | 4.41E-60 | 2.00E-57 |
| GLS | 1.749666 | -0.343976573 | 0.002758242 | 0.6910572 | 1.14E-29 | 1.42E-27 |
| STAT1 | 2.413008 | -1.080323786 | 0.003944816 | 0.24590035 | 6.08E-09 | 1.19E-07 |
| SF3B1 | 1.593996 | 2.124867831 | 1.31E-07 | 0.13815334 | 0.002676459 | 0.01368722 |
| TYW5 | 1.216825 | 0.809439436 | 0.002123855 | 0.31859086 | 0.008686158 | 0.03658756 |
| SPATS2L | 1.150042 | 0.24165084 | 0.01564512 | 0.39149589 | 1.28E-09 | 2.79E-08 |
| BZW1 | 1.215818 | 0.504066029 | 0.00525818 | 0.53934957 | 2.17E-33 | 3.11E-31 |
| NRP2 | 1.697439 | -0.380304322 | 0.004015236 | 0.23059825 | 0.001545753 | 0.008664171 |
| CCNYL1 | 1.793243 | -0.022850987 | 0.000612799 | 0.323759 | 3.52E-05 | 0.000327317 |
| ACSL3 | 1.312308 | 0.759664444 | 0.001157928 | 0.14211897 | 0.000346907 | 0.002445261 |
| KCNE4 | 1.568356 | 0.264082477 | 0.00108952 | 1.18218649 | 6.33E-16 | 2.93E-14 |
| SCG2 | 1.023716 | 0.379424457 | 0.0236259 | 1.86010234 | 9.46E-47 | 2.53E-44 |
| AGFG1 | 1.293718 | -0.021444844 | 0.01030639 | 0.24999391 | 1.27E-09 | 2.78E-08 |
| MLPH | 1.131098 | 0.379026348 | 0.01256134 | 0.278749 | 1.42E-09 | 3.08E-08 |
| LMCD1 | 1.237185 | 0.708804509 | 0.002466651 | 0.92750451 | 5.44E-21 | 3.62E-19 |
| PPARG | 1.32354 | 0.35635049 | 0.003978429 | 0.51008798 | 2.63E-09 | 5.53E-08 |
| ANKRD28 | 1.774005 | -0.174476339 | 0.001279284 | 0.34818845 | 8.93E-10 | 2.00E-08 |
| RARB | 1.047889 | 0.948948932 | 0.007641582 | 0.95079271 | 1.03E-13 | 3.79E-12 |
| RBMS3 | 1.38749 | 0.399781108 | 0.002254856 | 1.37783351 | 4.74E-07 | 6.64E-06 |
| CTNNB1 | 1.325705 | -0.342922401 | 0.01953014 | 0.13021141 | 0.005796614 | 0.02627209 |
| UCN2 | 1.448726 | 0.44193624 | 0.001266002 | 0.86236122 | 0.00695729 | 0.03044327 |
| PTP4A2 | 1.057665 | -0.172491634 | 0.04444927 | 0.13717812 | 0.000784092 | 0.004893534 |
| FLNB | 1.269401 | -0.379236848 | 0.02699405 | 0.17537805 | 9.89E-07 | 1.29E-05 |
| SUCLG2 | 1.045006 | 0.582618328 | 0.0133673 | 0.20303653 | 0.004637038 | 0.02170282 |
| FOXP1 | 2.41581 | 0.308286108 | 1.95E-06 | 0.62800482 | 0.00199074 | 0.01075974 |
| VGLL3 | 1.203876 | -0.079969011 | 0.01875381 | 0.43950745 | 8.01E-07 | 1.07E-05 |
| CBLB | 3.888543 | 0.770234917 | 1.20E-13 | 0.72905051 | 3.66E-18 | 2.02E-16 |
| BOC | 1.079117 | 0.544059234 | 0.01172047 | 1.00114605 | 2.83E-09 | 5.90E-08 |
| HEG1 | 1.365641 | 0.216715787 | 0.004704587 | 0.98052867 | 7.28E-42 | 1.50E-39 |

| **Table S5. Transcriptionally downregulated genes that had decreased H3K4me3 deposition within their promoter regions simultaneously in the CFP1-knockdown cell lines.** | | | | | | |
| --- | --- | --- | --- | --- | --- | --- |
| id | ChIP_seq | | | RNA_seq | | |
|  | log2FC | logCPM | PValue | log2FC | pvalue | padj |
| FLRT3 | -3.78172 | 0.3271149 | 1.59E-10 | -1.65275471 | 7.19E-60 | 3.17E-57 |
| PTGS2 | -1.892559 | 0.1271864 | 0.0003024 | -0.85836981 | 1.81E-57 | 7.57E-55 |
| IFNGR1 | -1.432046 | 0.5350208 | 0.0011292 | -0.58041753 | 3.37E-20 | 2.14E-18 |
| ITPRID2 | -1.747572 | 1.8322248 | 4.89E-08 | -0.28183447 | 5.86E-08 | 9.56E-07 |
| ANKRD22 | -2.375683 | 0.6150275 | 2.72E-07 | -1.75033908 | 2.95E-06 | 3.51E-05 |
| THBD | -1.250346 | 0.6114291 | 0.0034295 | -1.1594802 | 1.77E-45 | 4.39E-43 |
| FAM83A | -2.270048 | 0.4135711 | 2.80E-06 | -2.5068269 | 3.48E-90 | 3.79E-87 |
| EPS8 | -1.339388 | 1.0037759 | 0.0003477 | -0.37137433 | 2.17E-07 | 3.21E-06 |
| CPLX2 | -1.305552 | 1.1658206 | 0.000293 | -0.66634878 | 7.08E-77 | 4.63E-74 |
| MPRIP | -1.551075 | 1.4238528 | 7.24E-06 | -0.50359162 | 2.57E-51 | 7.91E-49 |
| DUSP16 | -1.436048 | 0.1258557 | 0.005121 | -0.57957417 | 7.22E-27 | 7.37E-25 |
| CNKSR2 | -1.571738 | -0.1009429 | 0.004744 | -0.68737308 | 6.42E-08 | 1.04E-06 |
| RASSF10 | -1.13506 | 1.4949938 | 0.0006812 | -0.59385115 | 0.0019567 | 0.01060099 |
| CHL1 | -2.383759 | -0.0990049 | 4.94E-05 | -4.81456126 | 6.27E-07 | 8.57E-06 |
| MAPK4 | -2.644017 | -0.3318536 | 5.25E-05 | -4.79268414 | 8.76E-18 | 4.72E-16 |
| RNF43 | -1.204954 | 1.2995566 | 0.000549 | -1.04704811 | 0.0016672 | 0.009278245 |
| MUC5AC | -2.142004 | 0.0467009 | 9.19E-05 | -0.99597425 | 3.91E-117 | 5.81E-114 |
| SYT6 | -1.257758 | 1.640607 | 0.0001153 | -0.82096418 | 8.48E-05 | 0.000707442 |
| IGFBP3 | -1.015345 | 2.7378626 | 0.0001285 | -1.6711139 | 1.67E-187 | 1.22E-183 |
| SCEL | -1.954652 | 0.0462429 | 0.0002966 | -2.31994225 | 9.39E-25 | 8.34E-23 |
| C1orf116 | -1.26202 | 1.5157955 | 0.0001603 | -1.69786941 | 1.49E-11 | 4.21E-10 |
| ARPC5 | -1.277572 | 1.4226512 | 0.0001788 | -0.3684487 | 1.44E-12 | 4.63E-11 |
| SMAD2 | -1.620286 | 0.5938848 | 0.0002107 | -0.27088039 | 8.20E-08 | 1.30E-06 |
| SMAD6 | -1.380539 | 1.0322749 | 0.0002137 | -0.70374127 | 9.60E-34 | 1.39E-31 |
| TGFBR3 | -1.711788 | 0.4119968 | 0.0002187 | -0.44177261 | 1.52E-06 | 1.91E-05 |
| PLA2G4A | -1.245906 | 1.4009477 | 0.0002694 | -0.86081469 | 4.71E-34 | 7.00E-32 |
| OAS1 | -1.412337 | 1.0040744 | 0.0002727 | -1.01554079 | 1.66E-53 | 5.88E-51 |
| SCARA3 | -1.391615 | 0.989614 | 0.0003477 | -0.49191769 | 8.77E-10 | 1.97E-08 |
| DUSP4 | -1.119738 | 1.7462943 | 0.0003664 | -0.33006612 | 1.87E-23 | 1.47E-21 |
| NTS | -1.293932 | 1.1137075 | 0.0003849 | -1.42989889 | 3.07E-57 | 1.25E-54 |
| SGCD | -1.650548 | 0.3679958 | 0.0004103 | -0.47648714 | 0.0021405 | 0.01144409 |
| LHX8 | -1.685265 | 0.2997972 | 0.0004187 | -1.19346499 | 0.0077629 | 0.03331341 |
| SLC23A1 | -1.393783 | 0.8847696 | 0.0004869 | -0.70261981 | 0.0072908 | 0.03167491 |
| DENND1A | -1.200911 | 1.3340664 | 0.0005212 | -0.35400751 | 3.36E-05 | 0.000313635 |
| FN1 | -1.248312 | 1.1267026 | 0.0005762 | -0.49965812 | 3.51E-54 | 1.30E-51 |
| RASSF9 | -1.768399 | 0.1528965 | 0.0006027 | -0.62765159 | 6.95E-08 | 1.12E-06 |
| NFKB2 | -1.410445 | 0.7204598 | 0.0007308 | -0.26834483 | 9.07E-07 | 1.19E-05 |
| PDZD8 | -1.654083 | 0.1781524 | 0.0008206 | -0.15656196 | 0.0016873 | 0.00937091 |
| NOTCH3 | -1.121398 | 1.4536631 | 0.0008683 | -0.60765664 | 4.41E-40 | 8.18E-38 |
| ARHGAP26 | -1.901821 | -0.1311771 | 0.0008835 | -0.39751393 | 9.72E-10 | 2.16E-08 |
| EVA1C | -1.158228 | 0.2016799 | 0.020365 | -0.64045889 | 2.92E-17 | 1.47E-15 |
| LMO7 | -1.111797 | 1.3107268 | 0.0013394 | -0.46255514 | 1.97E-15 | 8.66E-14 |
| FAM110B | -1.402972 | 0.5149838 | 0.0014896 | -2.08498451 | 0.0009418 | 0.005702776 |
| NUAK2 | -1.02547 | 1.5736035 | 0.0017692 | -0.33644114 | 1.67E-08 | 3.01E-07 |
| ROBO1 | -1.461166 | 0.3222083 | 0.0018017 | -0.26142569 | 3.33E-05 | 0.000311135 |
| DDIT4 | -1.011177 | 1.5926577 | 0.0019668 | -0.23242397 | 6.05E-07 | 8.30E-06 |
| PC | -1.32945 | 0.5346481 | 0.0023906 | -0.26864153 | 6.44E-05 | 0.00055447 |
| KIAA1522 | -1.343005 | 0.4740594 | 0.0025763 | -0.48178036 | 8.53E-15 | 3.52E-13 |
| TC2N | -1.541967 | 0.0996605 | 0.0029765 | -0.59177608 | 5.67E-11 | 1.47E-09 |
| PRR12 | -1.109287 | 0.9444243 | 0.0031209 | -0.46515919 | 1.60E-14 | 6.43E-13 |
| PFKFB3 | -1.021699 | 1.2870069 | 0.0032667 | -0.36611756 | 4.08E-17 | 2.04E-15 |
| PVR | -1.084853 | 0.9737964 | 0.0035845 | -0.19473936 | 6.80E-05 | 0.000582689 |
| ONECUT3 | -1.474396 | 0.1520186 | 0.0037784 | -3.41571693 | 1.53E-06 | 1.92E-05 |
| CD55 | -1.020617 | 1.2147785 | 0.0037998 | -0.73307472 | 3.36E-50 | 1.02E-47 |
| TGM2 | -1.269483 | 0.4942865 | 0.0040528 | -0.9934055 | 4.69E-184 | 1.91E-180 |
| C11orf86 | -1.061558 | 1.0025817 | 0.0040913 | -0.86965762 | 2.90E-07 | 4.18E-06 |
| PDLIM5 | -1.221847 | 0.5924326 | 0.0044065 | -0.1516468 | 0.0015066 | 0.00847376 |
| PPP1R37 | -1.221847 | 0.5924326 | 0.0044065 | -0.39172067 | 9.75E-07 | 1.28E-05 |
| HEATR5A | -1.280437 | 0.4319463 | 0.0044155 | -0.37415539 | 1.07E-05 | 0.000111873 |
| CPS1 | -1.062177 | 0.9590326 | 0.0044433 | -0.82547938 | 2.34E-56 | 8.89E-54 |
| OXSR1 | -1.292503 | 0.3667922 | 0.0048176 | -0.16622363 | 0.0125175 | 0.0494382 |
| NRARP | -1.238538 | 0.4736764 | 0.0052582 | -0.72414517 | 2.75E-06 | 3.30E-05 |
| EIF3CL | -1.238538 | 0.4736764 | 0.0052582 | -0.95518571 | 0.0037676 | 0.01819653 |
| AKR1C3 | -1.113943 | 0.8522126 | 0.0052691 | -0.63407906 | 1.41E-61 | 6.60E-59 |
| CUX1 | -1.017337 | 1.0166133 | 0.0057019 | -0.39375489 | 1.99E-16 | 9.53E-15 |
| ABTB1 | -1.248105 | 0.4104221 | 0.0057589 | -0.52873681 | 0.0001158 | 0.000925843 |
| NAB1 | -1.153452 | 0.6662674 | 0.0059933 | -0.46011831 | 5.44E-12 | 1.63E-10 |
| KRT8 | -1.039038 | 0.8987404 | 0.0059998 | -0.60519157 | 2.15E-83 | 1.76E-80 |
| LAMB3 | -1.163101 | 0.5536762 | 0.0072237 | -0.40338316 | 3.48E-10 | 8.27E-09 |
| LTBR | -1.174582 | 0.4315562 | 0.0087815 | -0.26858067 | 1.46E-08 | 2.66E-07 |
| KIFC3 | -1.356151 | 0.07207 | 0.0093154 | -0.41644126 | 1.64E-23 | 1.31E-21 |
| C1QTNF1 | -1.097005 | 0.6294661 | 0.0095716 | -0.31746912 | 9.13E-06 | 9.74E-05 |
| TNRC18 | -1.181182 | 0.3663909 | 0.009715 | -0.506325 | 1.77E-20 | 1.14E-18 |
| PI3 | -1.181182 | 0.3663909 | 0.009715 | -2.16289399 | 1.28E-27 | 1.38E-25 |
| MYRF | -1.038692 | 0.7533696 | 0.0111375 | -0.43302025 | 9.10E-13 | 3.02E-11 |
| PRAG1 | -1.141509 | 0.4100283 | 0.0113003 | -0.47700355 | 8.21E-15 | 3.42E-13 |
| DUT | -1.101861 | 0.5138555 | 0.0117205 | -0.21719522 | 0.0072862 | 0.03167028 |
| HHIPL2 | -1.067931 | 0.6107091 | 0.0120505 | -0.27745642 | 0.0007049 | 0.004476536 |
| ASS1 | -1.31448 | 0.0444105 | 0.0124967 | -0.30086624 | 4.04E-05 | 0.000370003 |
| TOMM40 | -1.104626 | 0.4523827 | 0.013018 | -0.23375802 | 3.55E-07 | 5.06E-06 |
| CITED2 | -1.069035 | 0.5533067 | 0.0133673 | -0.4095931 | 7.55E-06 | 8.17E-05 |
| TMTC1 | -1.069035 | 0.5533067 | 0.0133673 | -1.53557681 | 0.0078001 | 0.03342414 |
| LIMCH1 | -1.038415 | 0.6476295 | 0.013628 | -0.17734722 | 0.0002331 | 0.001723397 |
| UBL4A | -1.038415 | 0.6476295 | 0.013628 | -0.29225987 | 2.18E-05 | 0.000213342 |
| B4GALT5 | -1.333262 | -0.0414066 | 0.013932 | -0.39234243 | 1.62E-17 | 8.41E-16 |
| SLC39A8 | -1.152063 | 0.2745211 | 0.0139996 | -0.49405562 | 1.73E-14 | 6.92E-13 |
| MTUS1 | -1.10766 | 0.3881761 | 0.0144973 | -0.3030414 | 9.99E-12 | 2.90E-10 |
| TNFRSF19 | -1.10766 | 0.3881761 | 0.0144973 | -1.80145009 | 6.89E-08 | 1.11E-06 |
| PCIF1 | -1.10766 | 0.3881761 | 0.0144973 | -0.27941371 | 0.0011265 | 0.006654086 |
| PRELID1 | -1.257479 | 0.0987638 | 0.014908 | -0.49563999 | 9.48E-12 | 2.77E-10 |
| LSM4 | -1.257479 | 0.0987638 | 0.014908 | -0.47636145 | 2.32E-14 | 9.20E-13 |
| ZNRF3 | -1.010611 | 0.6836275 | 0.01531 | -0.69002736 | 2.76E-25 | 2.54E-23 |
| TBL1X | -1.111004 | 0.3209811 | 0.0161891 | -0.29110291 | 7.90E-06 | 8.53E-05 |
| CLTA | -1.205628 | 0.1511407 | 0.0175335 | -0.20549165 | 6.28E-05 | 0.000541761 |
| CDC34 | -1.215808 | 0.0716168 | 0.0197076 | -0.26450673 | 0.0002397 | 0.001762418 |
| FDX1 | -1.037704 | 0.4096345 | 0.0210435 | -0.41054798 | 2.17E-05 | 0.000211829 |
| MN1 | -1.037704 | 0.4096345 | 0.0210435 | -1.04502778 | 2.29E-18 | 1.27E-16 |
| DCBLD1 | -1.006467 | 0.5134793 | 0.0211578 | -0.39991468 | 2.82E-07 | 4.09E-06 |
| GLIS3 | -1.037482 | 0.3434585 | 0.0236259 | -0.24672961 | 0.000652 | 0.004190522 |
| ARMC5 | -1.037482 | 0.3434585 | 0.0236259 | -0.38915925 | 0.0099891 | 0.04116959 |
| ID3 | -1.004832 | 0.4519962 | 0.0236976 | -0.28285602 | 5.57E-08 | 9.13E-07 |
| PCDHAC2 | -1.172897 | 0.0439523 | 0.0259426 | -1.01552783 | 3.38E-10 | 8.05E-09 |
| OGDH | -1.128671 | 0.0157501 | 0.0339961 | -0.13154687 | 0.0091224 | 0.03814006 |
| PPP1CA | -1.080544 | 0.0711636 | 0.0388023 | -0.23594058 | 4.18E-06 | 4.76E-05 |
| RNF19B | -1.03666 | 0.1245247 | 0.0438256 | -0.5255807 | 1.93E-08 | 3.43E-07 |

**Table S6. The GO enrichment analysis of genes displaying downregulated expression and less H3K4me3 modification in RNA-seq and ChIP-seq.**

| Pathway | PValue | Genes | Fold Enrichment |
| --- | --- | --- | --- |
| Wnt receptor catabolic process | 0.0096 | RNF43, ZNRF3 | 205.9255 |
| cellular response to oleic acid | 0.0143 | CPS1, ASS1 | 137.2837 |
| zygotic specification of dorsal/ventral axis | 0.0143 | SMAD2, SMAD6 | 137.2837 |
| cellular nitrogen compound metabolic process | 0.0002 | PC, CPS1 | 123.5553 |
| cellular response to ammonium ion | 0.0238 | CPS1, ASS1 | 82.3702 |
| 'de novo' pyrimidine nucleobase biosynthetic process | 0.0332 | CPS1 | 58.8359 |
| negative regulation of platelet activation | 0.0378 | THBD, C1QTNF1 | 51.4814 |
| midgut development | 0.0378 | CPS1, ASS1 | 51.4814 |
| positive regulation of synaptic plasticity | 0.0378 | CPLX2, PTGS2 | 51.4814 |
| cyclooxygenase pathway | 0.0424 | AKR1C3, PTGS2 | 45.7612 |
| positive regulation of cellular respiration | 0.0424 | PRELID1, OAS1 | 45.7612 |
| pulmonary valve morphogenesis | 0.0030 | SMAD2, SMAD6, ROBO1 | 36.3398 |
| peptide cross-linking | 0.0091 | FN1, PI3, TGM2 | 20.5926 |
| aortic valve morphogenesis | 0.0129 | SMAD2, SMAD6, ROBO1 | 17.1605 |
| ventricular septum morphogenesis | 0.0136 | TGFBR3, CITED2, ROBO1 | 16.6967 |
| dephosphorylation | 0.0259 | DUSP4, DUSP16, PPP1CA | 11.8803 |
| cellular response to cAMP | 0.0307 | CPS1, FDX1, ASS1 | 10.8382 |
| liver development | 0.0065 | TGFBR3, DUT, CITED2, ASS1 | 10.4266 |
| TGF beta receptor signaling pathway | 0.0121 | TGFBR3, SMAD6, CITED2, SMAD2 | 8.3202 |
| response to lipopolysaccharide | 0.0311 | THBD, CPS1, SMAD6, PTGS2 | 5.8007 |
| heart development | 0.0186 | CITED2, FLRT3, FN1, ID3, PDLIM5 | 4.9030 |
| negative regulation of gene expression | 0.0167 | SMAD2, PC, CITED2, ID3, NTS, ROBO1 | 3.9986 |
| ubiquitin-dependent protein catabolic process | 0.0476 | UBL4A, RNF43, ZNRF3, CDC34, RNF19B | 3.6383 |
| positive regulation of gene expression | 0.0118 | SMAD2, C1QTNF1, CITED2, IFNGR1, FN1, ID3, NTS, ROBO1 | 3.2113 |
| intracellular signal transduction | 0.0214 | CNKSR2, SMAD2, TGFBR3, NUAK2, OXSR1, PDZD8, MAPK4 | 3.2033 |
| nervous system development | 0.0485 | RASSF10, FN1, ARHGAP26, CPLX2, PCDHAC2, ROBO1 | 2.9989 |
