# Supplementary material for: CXXC finger protein 1 (CFP1) bridges the reshaping of genomic H3K4me3 signature to the advancement of lung adenocarcinoma
Source: Signal Transduct Target Ther. 2023 Sep 21;8:369. doi: 10.1038/s41392-023-01612-3 (PMC10514036; doi:10.1038/s41392-023-01612-3)

Fig. 4a

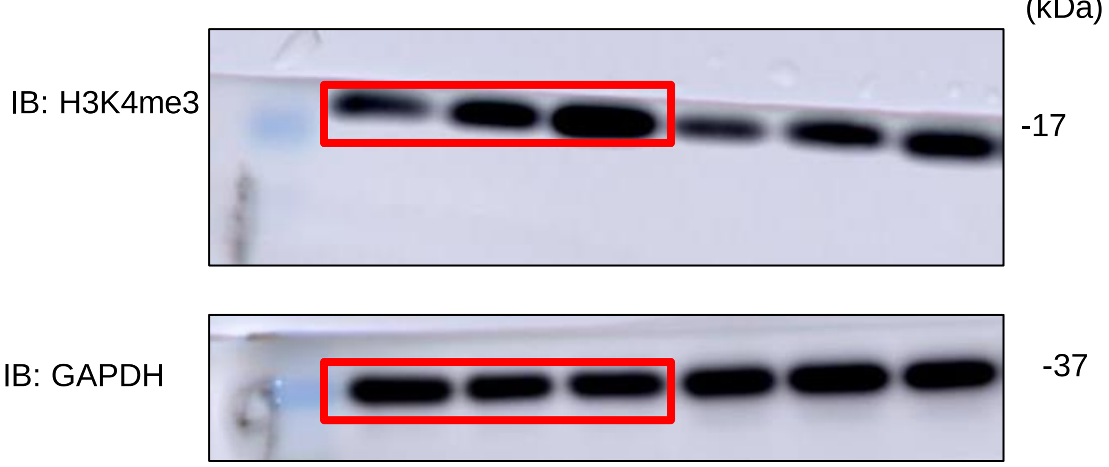

Fig. 4g

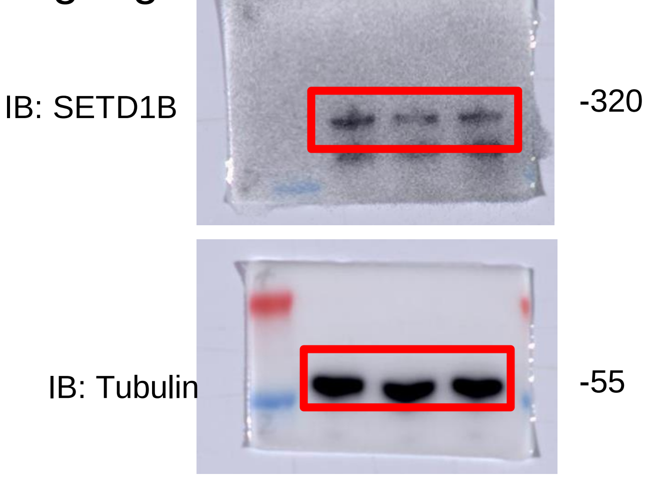

Fig. 4i

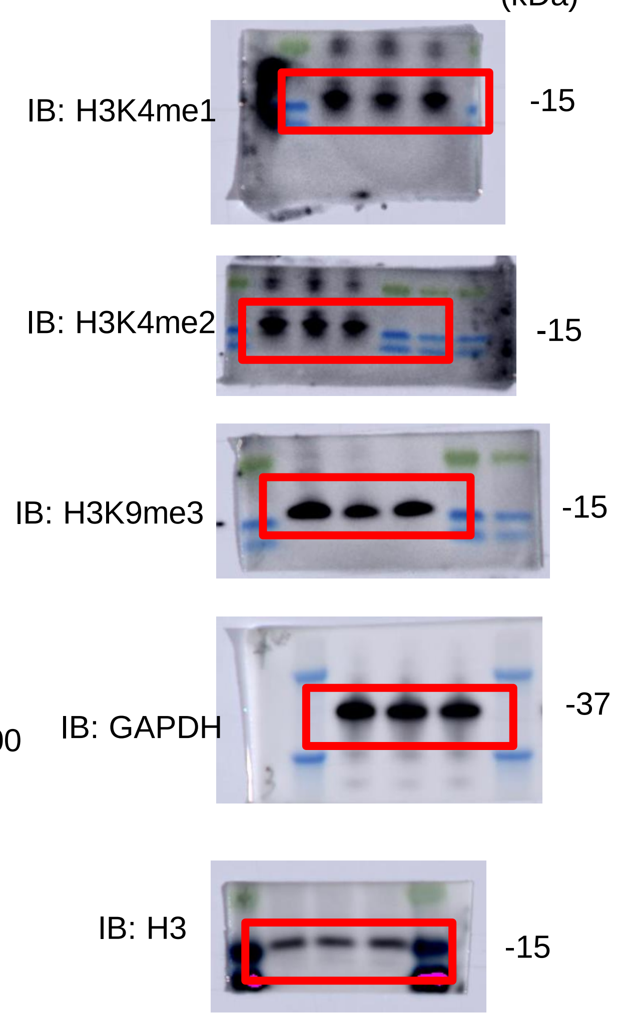

Fig. 4b

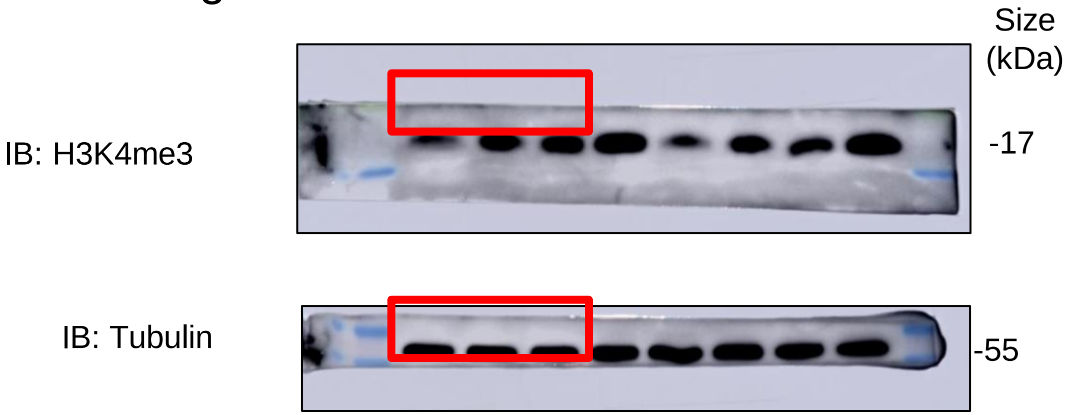

Fig. 4h

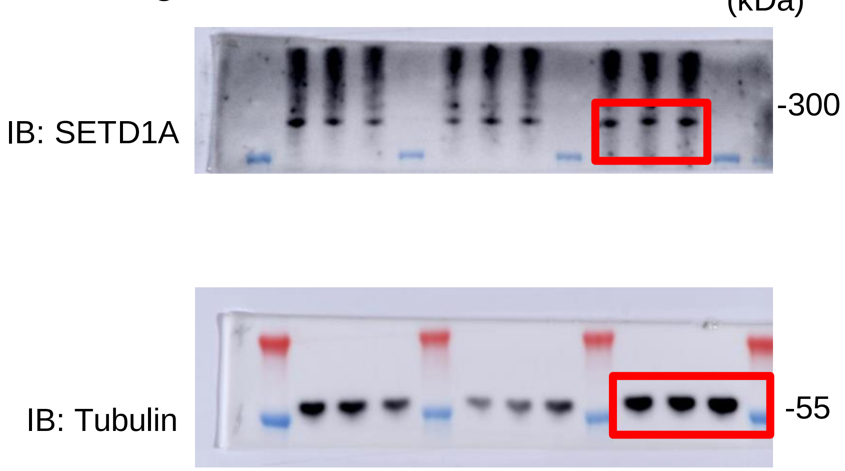

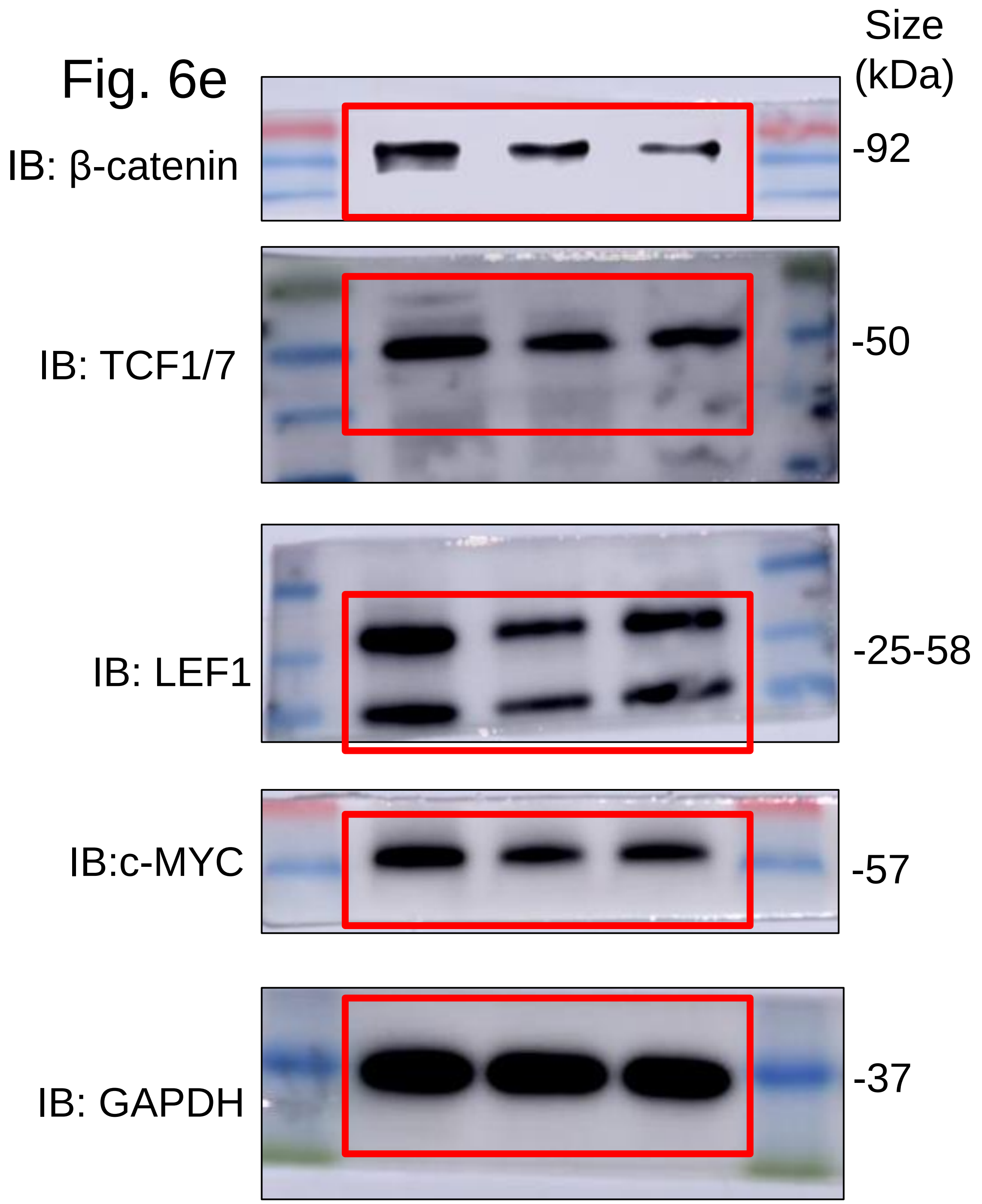

Fig. 6e
Size (kDa)
IB: β-catenin
-92
IB: TCF1/7
-50
IB: LEF1
-25-58
IB:c-MYC
-57
IB: GAPDH
-37

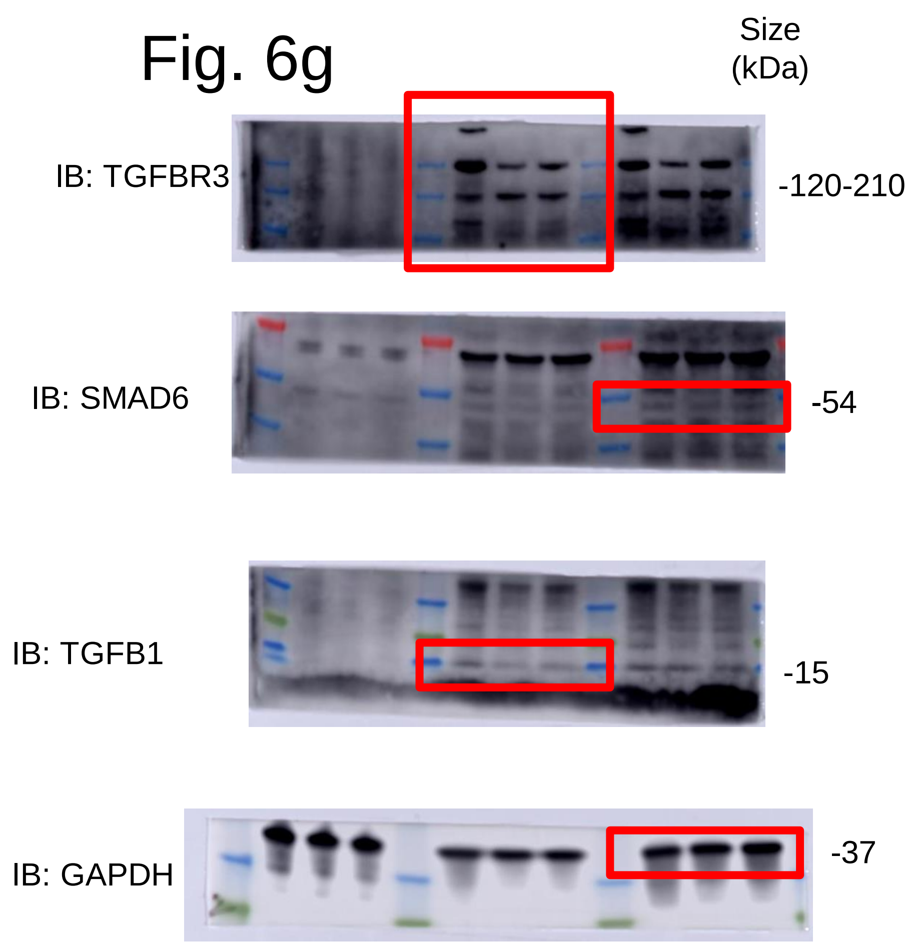

Fig. 6g
Size (kDa)
IB: TGFBR3
-120-210
IB: SMAD6
-54
IB: TGFB1
-15
IB: GAPDH
-37

Fig. 6i

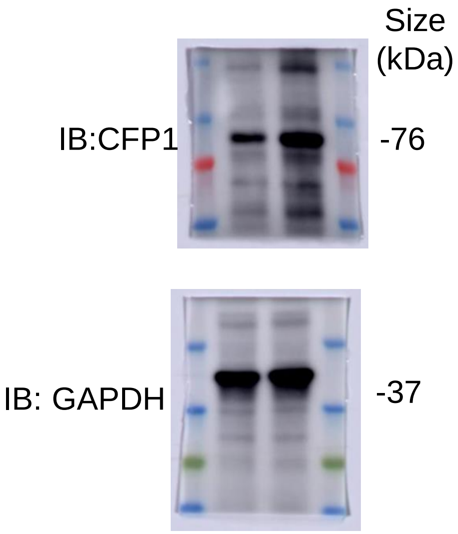

Fig. 6p

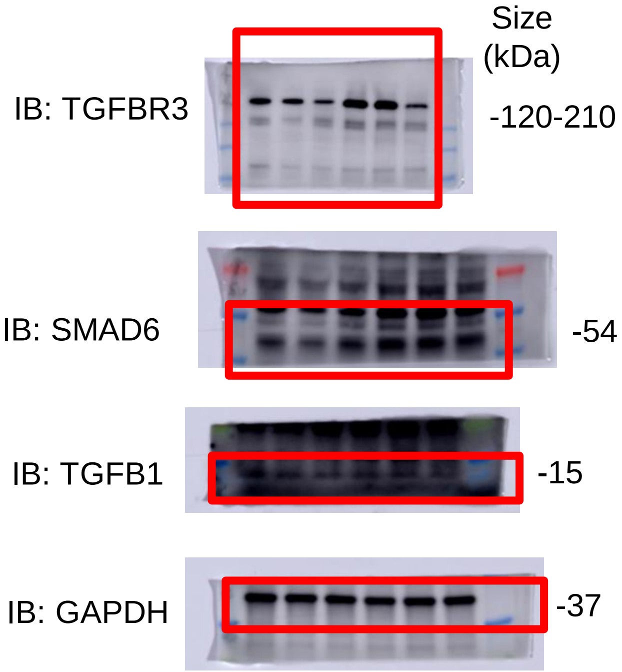

Fig. 6q

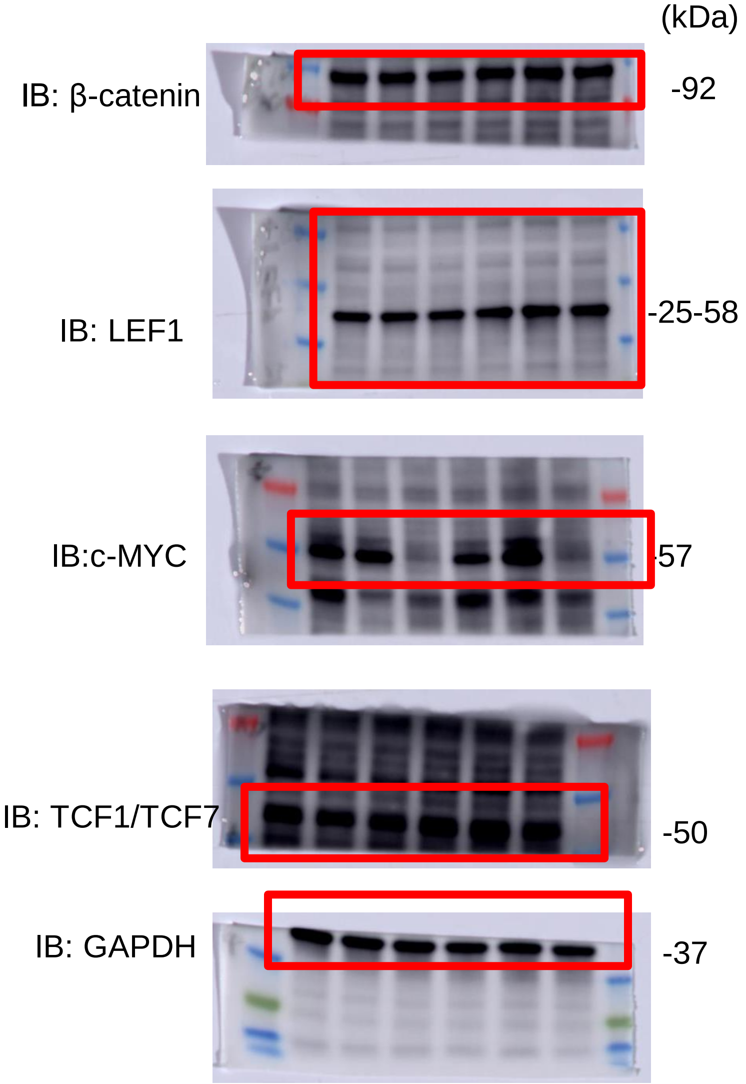

Supplemental figure 2a

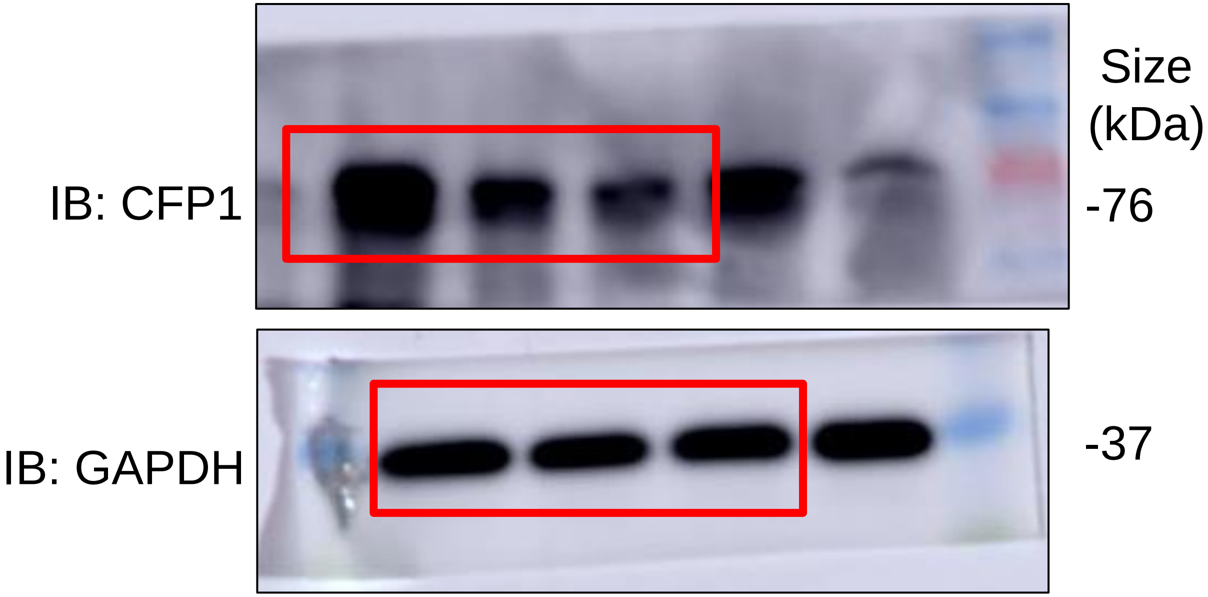

Supplemental figure 2b

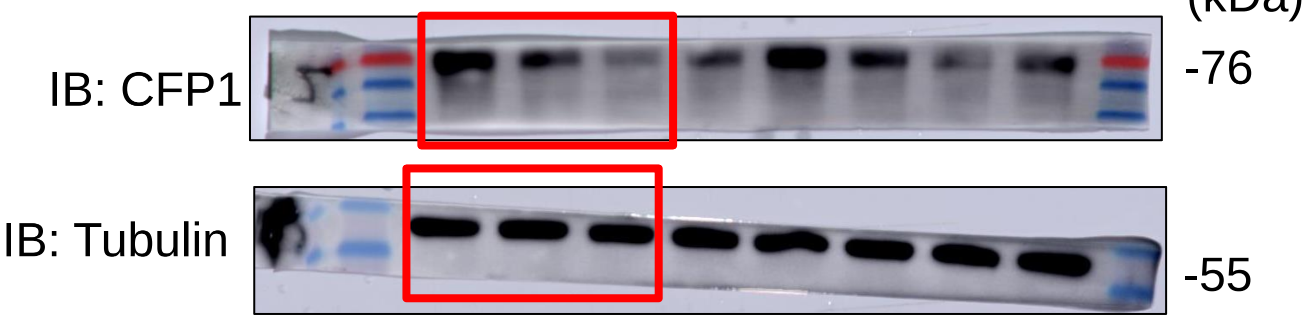

Supplement: Supplementary file 2 — Original western blot results [file 41392_2023_1612_MOESM2_ESM.pdf]
